# Investigation of crack initiation, and mechanical behavior of polypropylene and polymer-based composites under plastic deformation in automotive brake fluid reservoirs

**DOI:** 10.1371/journal.pone.0352763

**Published:** 2026-09-18

**Authors:** Liang Jian Chun, Ying Quanjia, Yang Bin, Zhang Debing, Zhou Sen, Kefu Wang, Asif Raza, Malik Haris, Jibran Hussain

**Affiliations:** 1 Ningbo Qiaoshi Rubber and Plastics Co., Ltd., Ningbo, China; 2 Zhejiang Qiaoshi Industry Co., Ltd., Ningbo, China; 3 School of Information Science and Engineering, NingboTech University, Ningbo, China; 4 School of Control Science and Engineering, Tianjin University, Tianjin, China; National Textile University, PAKISTAN

## Abstract

This paper discusses the intensive research on polypropylene, a significant polymeric material, and its extensive application in the automotive industry, particularly in the manufacture of brake fluid reservoirs through a series of laboratory destruction tests and experiments, The paper emphasizes the triaxiality (Triax) (η = σm/σeq) is used as the theoretical framework for evaluating crack initiation and behavior under various stress states and critical factors for the understanding of crack formation intention and the mechanical behavior of polypropylene under mechanical loads. This study present a detailed evaluation of polypropylene behavior under various manufacturing conditions, addressing potential deviations that could affect final product quality. The findings demonstrate the material’s robustness and high fracture toughness, thermal stability, and mechanical strength minimize crack initiation, leakage, damage, and other defects during the manufacturing process, ensuring reliable use in reservoir production. The research methodology involved a series of tests and experimental procedures, such as (i) Melt Flow Index (MFI = 0.109 g/10 min, ASTM D1238, 230°C/5 kg); (ii) uniaxial tensile testing (yield strength: 23.0–23.7 MPa, tensile modulus: 2.36–2.48 GPa, fracture strain: 42–68%, ASTM D638, n = 3); (iii) burst pressure testing (mean burst: 2.70 ± 0.10 MPa at Δp = 11 bar/s, minimum requirement: 0.70 MPa, ISO 8033); (iv) air leakage testing (no leakage at 500–700 kPa hold for 60 s); (v) thermal chamber cycling (−40°C to 120°C, 95% RH, 24 h; dimensional change < 0.02 mm); (vi) tilt alarm test (alarm activation at ≤ 0.53° tilt); and (vii) volume capacity verification (within OEM specification) during manufacturing of the brake fluid reservoir. These results confirm the long-term suitability of SABIC 83MF10-polypropylene for safety-critical reservoir applications in modern automotive braking systems. The novel aspects of this study are the demonstration of the mechanical behaviour of polypropylene and the valuable insights provided into the brake fluid reservoir manufacturing process and performance, offering significant implications for automation in the automotive sector.

## 1. Introduction

**Fig 1:**
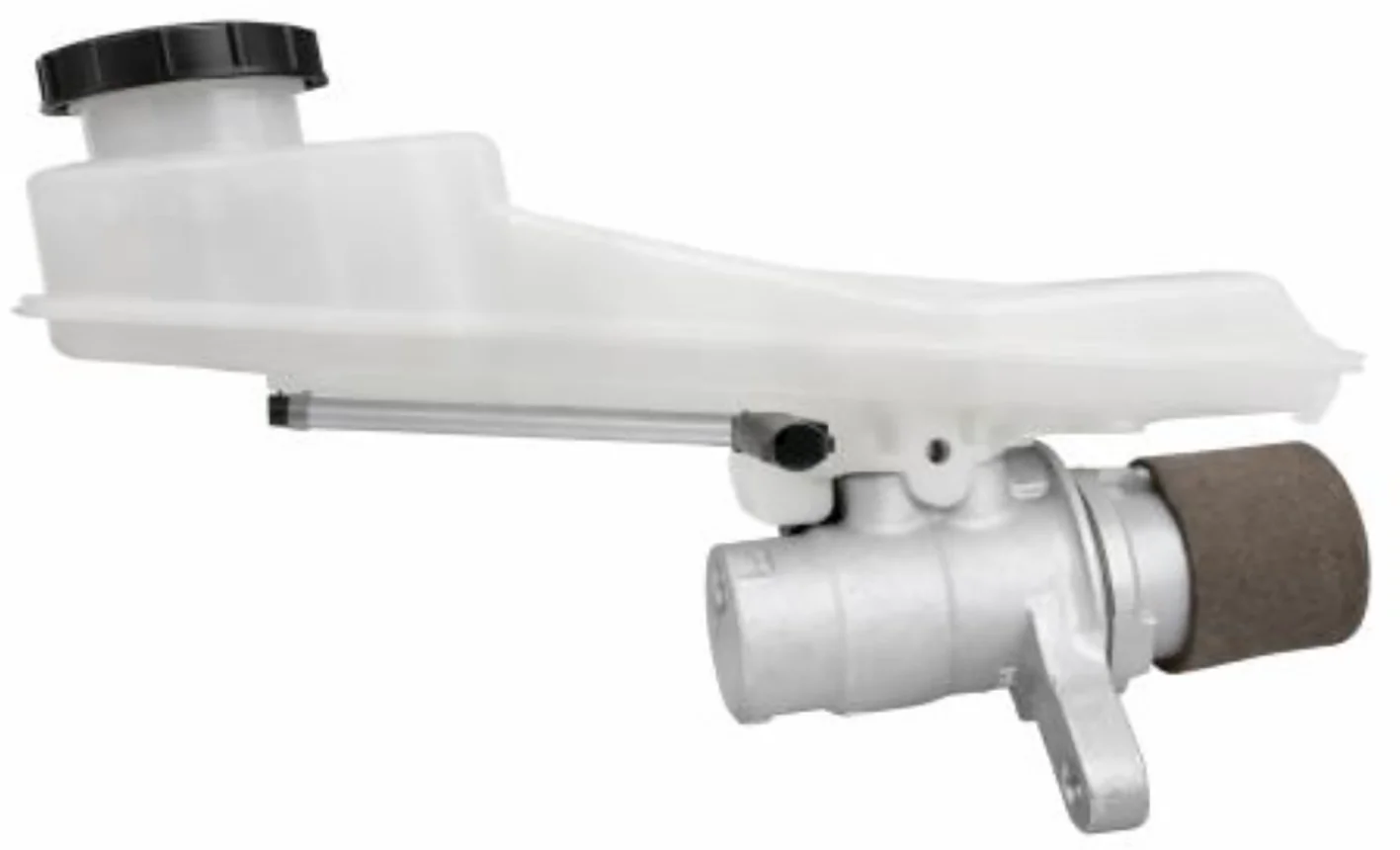
Brake fluid reservoir placed on master cylinder.

Polymer-based materials, particularly polypropylene, which was developed in 1951 [1], marked a significant breakthrough in the field of materials science and engineering. These materials are now being used in industries through different applications. Polypropylene has excellent mechanical properties, thermal stability, and chemical resistance, making it an ideal material for polymers [2,3]. To maintain dimensional stability throughout its life cycle, superior resistance to crack initiation, leakage, damage, and most importantly, its suitability for high-volume production processes such as injection molding [4,5]. Due to these excellent qualities, polypropylene has found extensive application in the automobile industry; it is not only used in the interior design of vehicle fabrication but also in the production of parts, and it helps reduce mechanical stress and improves vehicles safety [6]. Specifically, the brake fluid reservoir is a critical part of the braking system and is used in both conventional vehicles and modern electric vehicles (EVs). Its primary function is to store brake fluid and transmit it to the master cylinder and the entire hydraulic braking system of the vehicle [7]. Brake fluids, such as DOT 3, DOT 4, and DOT 5.1, are glycol-based and gyroscopic, which means that they naturally absorb moisture from the air [8]. This contamination may lower the boiling point of the fluid with time [9]. In extreme conditions, such as high temperature, pressure, and cold weather, the reservoir tank can be damaged, cracked, and leak, which may affect braking efficiency and safety. Therefore, the reservoir tank should have good resistance to cracking, damage, leakage and, and remain usable for a long time. Fig 1 shows the brake fluid reservoir that is placed in the vehicles braking system was produced by the company.

This study investigates the method for crack initiation and material behavior of polypropylene in automotive brake fluid reservoir applications is crucial for improving the stability and suitability of polypropylene in the automotive industry. Previously studies conducted these tests to determine the polymers materials parameters within the focus on influence of triaxiality on the cracking and these experiments mainly employed printed samples to permit specially prepared forms, thus permitting a comparatively broad variety of other material stress conditions, known as TRIAX (triaxiality) [10–15]. Another study author proposed a peak parameter on the basis of triaxiality of the crack initiation during the manufacturing of the reservoir with plastic strain-dependent factors and triaxiality predication to predict cracks formed in the polypropylene under pressure loading, and reports the change of material strain according to triaxiality conditions with the plastic strain (PEEK) and plastic strain energy to measure the crack initiation. The results confirm that the peak parameter significantly improves component design and material selection. Consequently, in recent years, researchers have used numerous methods to predict crack initiation and the material behavior of polypropylene, such as Finite Element Method (FEM) and Differential Element Method (DQM) and Bezier Multi-step method. The correlation between the structure and properties of isotactic polypropylene in opposing processing conditions has been extensively examined [16]. This study, compression-molded samples were subjected to two vastly different cooling conditions such as, rapid quenching and gradual cooling, representing extreme conditions in industrial processing. Although the source was the same commercial polypropylene with high isotactic content, significant differences in crystalline structure, morphological characteristics, and physical behavior, especially mechanical performance, were reported. The authors in [17] have been reviewed the impact of decomposition polypropylene on environmental toxicity, which poses significant ecological and toxicological risks. The reviewed studies have shown that polypropylene accumulates in ecosystem and degrades into micro plastics, which can harm organism and disrupt ecological process, identifying microorganism and enzymes capable of initiating polymer break down, offering promising alternative for plastic waste management.

Similarly, a comprehensive review on application and research of polypropylene is provide in [18] to examine the recent development based on advance applications and nano materials. This study has also identified various constraints on applications in automotive, aerospace, and filtration through a critical assessment of various fabrication techniques. Furthermore, current research effects aim to address these challenges and further topics are also discussed in detail. However, there are also significant research gaps that should be filled in through further research. In [19], authors have been investigating how the water content in brake fluid influences the rate at which break temperature rises and its boils point. This study concludes that the moisture level in brake fluid impacts both the temperature increases rate and boiling point. As water content in the brake fluid increases, the temperature achieved by the brake fluid decreases, and its boiling point also lowers. Similarly, the impact of brake fluid on the efficiency of the braking system is determined in [20] by conducting tests on brake discs of a laboratory vehicle heated to approximately 400°C. During these tests, the brake fluid temperature was monitored, and the boiling points of brake fluid within test vehicles brake system were measured. These findings were contrasted with brake fluid samples of various factors, which were at the same level of specification, to investigate differences in performance. Consequently, the study is significant in enhancing the efficiency of the brake system, safety, as well as informing the development of superior technologies of brake fluids. Another study has been studies in injection molding parameters on the polypropylene properties [21], which is focusing on such factors, melt injection distances, tool surface, processing conditions, and also varying the injection molding pressure 20–80 Mega pascal (MPa) and mold temperature about 20–60 °C, which directly influences the mechanical properties and crystalline of the molded parts. They found that a maximum of 27% improvement in injection pressure produced an improvement in mechanical properties, with mold temperatures of 40°C and higher producing a maximum improvement of 17%. These changes in injection molding pressure and the temperature was very close and associated with variations in crystallinity and their impact on the mechanical properties. The literature review concluded that polypropylene predicting crack initiation based on material behaviour properties, stress conditions, and triaxiality factors. The absence of failure in these critical tests provides strong confidence in the long-term reliability of the reservoirs tank, ensuring the safety and performance of the vehicles. The current study focuses on the traiaxiality testing and examination the material response stress states ranging from shear to traiaxial stretching, and demonstrated a distinctly material behavior, mechanical stress under different physical test provides real world validation of theoretical model and bringing the gap of simulations and practical material behavior. This study reports no premature crack formation, damage and leakage were observed, which is evidence of polypropylene exhibiting higher resistance and high-quality for the manufacturing of the reservoir.

Therefore, this research represents a novel contribution that provides practical guidelines and scientific insight. The current study involved the multi-test during the production line and general validation of the SABIC 83MF10-polypropylene material used in the brake fluid reservoir manufacturing process, as well as investigating the mechanism and optimization of the design support and the reliability of the product to improve the safety of the braking system in modern vehicles. Specifically, the novel scientific contributions of this work are; (1) a fully physical verification of the triaxiality dependent crack resistance of SABIC 83MF10-polypropylene under the actual production conditions, closing the gap between the PEAK parameter prediction and the component actual performance;(2) demonstration of the material will not prematurely initiate a crack under loading rate of Δp = 11 and (3) determination of material behavior in multiaxial stress states over a complete set of qualification tests, a limitation of the present study is SABIC 83MF10-polypropylene was investigated and its grades which are easily processable and chemically compatible with the DOT 4 glycol based fluid than polymide and HDPE material used in brake fluid reservoir applications, these physical test are especially use full when selecting material in the the automotive braking system.This study differentiates itself from prior simulation-only work [16] by providing physical multi-test validation of the SABIC 83MF10 material under actual OEM production conditions, rather than virtual predictions alone. The remaining parts of this paper are structured as follows: Section 2 describes the material selection. Section 3 presents manufacturing process of the reservoir. Section 4 provides the result and discussion, and Section 5 outlines the conclusion.

## 2. Material selection

Brake fluid reservoir is usually made of polymers, specially polypropylene, which is the best choice due to the excellent chemical resistance, process ability, mechanical strength, and cost-effectiveness [22]. It is a versatile material widely used in automotive industry and brake fluid reservoirs due to its low density, high resistance to brake fluid, and good adhesion to hot plate welding, polypropylene inherently has a semi-crystalline structure with the formula of (C3H6) n strong carbon bonds [23,24] and the presence of a methyl group in the backbone, which gives it superior stiffness and rigidity properties [25]. It has good heat resistance and outstanding toughness [26], isotactic pp melting point is 171 °C and commercial isotactic melting point of 160–166°C which is depending on the atactic pp material and Crystallinity and syndiotactic with a Crystallinity rate is about 30% has 130 melting point °C [27,28]. It plays a significant role in the production of thermoplastic parts due to its durability, lightweight, and unique properties.

### 2.1. Basic material properties of polypropylene

SABIC 83MF10-polypropylene is widely used in the thermoplastic industry due to its excellent material properties and characteristics, such as a very high impact strength even at very low temperatures, combined with high stiffness and moderate flow properties, such as strength, flexibility, chemical resistance, and strong mechanical performance. It has superior impact resistance and structural integrity, making it perfect for the manufacturing of brake fluid reservoirs, and this material is very useful for injection molding [29,30]. This work will ensure the comparability of prior studies and the material evolution. The following Table 1 provides a detailed overview of the properties of polypropylene.

**Table 1:** Properties of polypropylene [31].

Property	Value
**Tensile strength**	3200-5000 psi (29–34 MN/m^2^)
**Elongation**	3-700%
**Softening point**	140-150°C
**Melting point**	160-166°C
**Flexural modulus**	160000,190000 psi (1100–1310 MN/m^2^)
**Water absorption**	0.01%
**Low moisture absorption**	0.02%
**Rockwell hardness**	90-95

## 3. Manufacturing process of the reservoir

**Fig 2:**
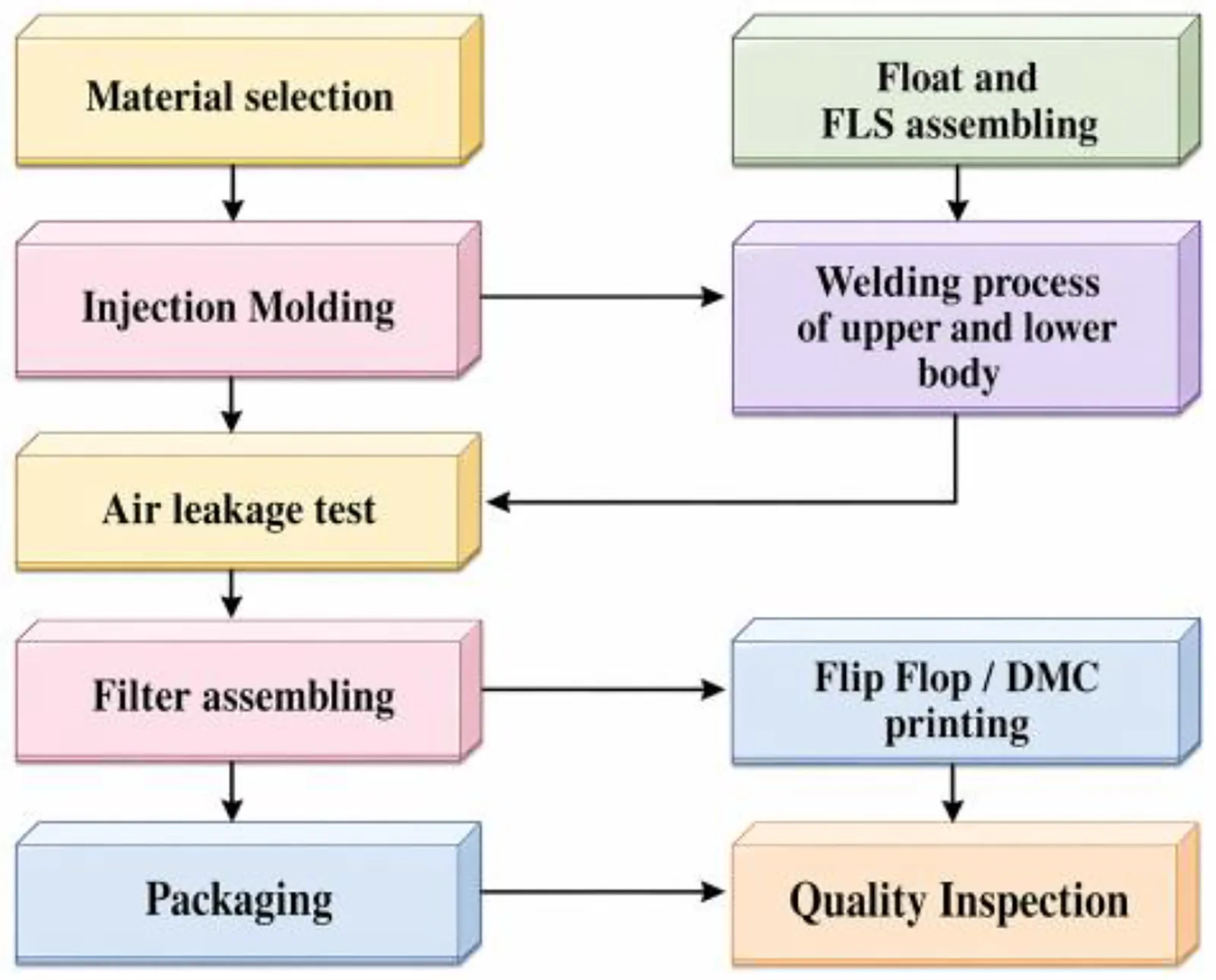
Flow chart process of manufacturing.

The manufacturing process starts with the selection of small polypropylene pellets. These pellets are placed into the injection molding machine through the hopper. The hopper is located on top of the injection molding machine. Next, the raw material is melted and injected into the mold to form the first shape of the reservoir. After the injection process, hot plate welding is carried out. Before welding, a magnetic float and fluid level sensor (FLS) is assembled into the reservoir. A flip-flop rotating check of the reservoir is then performed, allowing multi-sided access. After that, a net filter is installed, an air leakage test is done, and all manufactured products are marked using a laser data matrix code (DMC). Finally, inspection, quality assurance, and packaging are completed. Fig 2 shows the flowchart of the manufacturing process.

### 3.1. Manufacturing steps of the reservoir

This manufacturing process consists of three main stages, namely the injection molding workshop, the small parts workshop, and the assembly workshop. The injection molding workshop produces the main components of the reservoir tank, such as the upper and lower body parts are made in the injection molding workshop. The small parts workshop produces small accessories for the reservoir tank. In the assembly workshop, the upper and lower body parts of the reservoir tank are welded into a sealed tank. Finally, accessories are assembled in the reservoir tank to complete the production, inspection, and packaging to ensure all standards are met.

### 3.2. Raw material

This process begins with a selection of high-quality, superb SABIC 83MF10 polypropylene thermoplastic raw material and material class of polypropylene, the industry-standard choice for DOT 4 fluid compatibility. This grade was identified as optimal due to its combination of high impact strength at low temperatures, semi-transparency for fluid level visibility, high stiffness, and proven injection moldability. This work was aimed at validating the behavior of the real production material in the brake fluid reservoir during the respective OEM loading conditions. The SABIC 83MF10-polypropylene has excellent properties such as density, tensile modulus, Poisson ratio, and yield stress. Which ensure the SABIC 83MF10-polypropylene is an excellent material for the reservoir tank production, without reinforce inclusion, such as glass fiber, etc. Table 2 mentioned the basic material properties.

**Table 2:** Properties of SABIC 83MF10-Polypropylene [16].

Parameters	Value
**Poisson Ratio**	0.4 [−]
**Tensile Modulus**	1200 [MPa]
**Density**	0.905 [Kg/m^3^]
**Yield stress in RT**	25 [MPa]

### 3.3. Injection molding

Injection molding is the most common method for producing produce high-volume, complex, and precision plastic-shaped components [32]. Over the past 150 years, the automobile industry has made extensive use of injection molding technology to produce interior panels, bumpers, brake fluid reservoirs, and other plastic and automotive components for vital performance [33]. Injection molding is mostly used in polymer material pallets for the development of the products [34]. The majority of injection molding machines are screw-type, and injection molding is divided into two groups based on the operating mode: hydraulic and electric [35].

### 3.4. Injection molding working process

**Fig 3:**
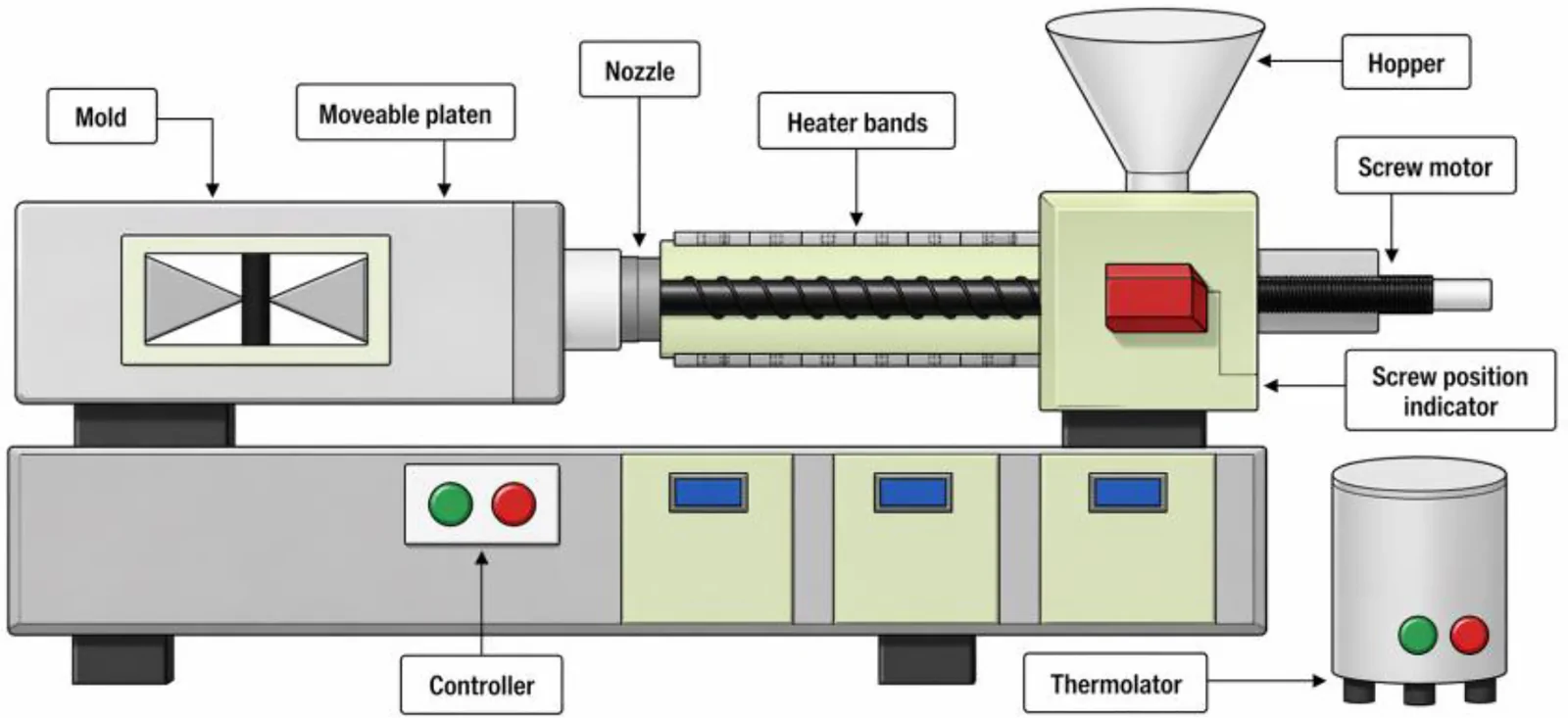
Injection molding working process [36].

An injection molding machine works by heating the material and using a rotating screw to inject it into a mold. It can be combined with external heaters, then inject molten plastic into the mold cavity, where it cools and solidifies into the desired shape. When this process is completed, the mold is separated into two parts, and the mold is opened. Fig 3 demonstrates how the injection molding machine works.

Initially, the material is dried, and then residual moisture is removed, which can damage the molding process. Then, the injection machine hopper is loaded with small pallets of raw polypropylene. These pallets are then fed into a barrel or hopper, which is heated by external heaters. The barrel subsequently mixes the polypropylene pallets through a rotating screw that is powered by an electrical motor. The screw position shows how the screw is moving within the monitor as a way of displaying accurate injection of molten plastic. After pushing melted plastic through the nozzle into the mold, which shapes the final product. The mold goes between two platens, which open and close during each molding cycle and are supported by tie bars. It helps with the tightness and closure of the mold in the injection process by adsorbing the clamping force and offering alignment. This controlling machine is the main interface that is operated by the operator, enabling the adjustment of the most important parameters, such as pressure, temperature, and time of cycling. A thermolator circulates and regulates oil through the mold to maintain the mold temperature and ensure consistent cooling. This ensures that the mold will remain at the ideal temperature and that plastic components are manufactured accurately and consistently and continuously monitored their parameters during production [37]. Polypropylene is the most widely used material in injection molding [38]. Because the polypropylene has very low viscosity during the molten state, it is feasible to produce a smooth and fluid consistency. It is easy to shape and can be very easily molded. It is an erosion, rust, and chemical spill-resistant material [36,39,40].

### 3.5. Injection molding parameters

The YIZUMI FF Series hydraulic injection molding machine (model FF90) was used in the production line. The key operational parameters employed during the injection molding process are detailed in Table 3.

**Table 3:** Injection molding parameters.

Parameter	Cycling time Value
**Purge**	38.8 sec
**Injection**	63.2 sec
**Mold Close**	4.3 sec
**Cooling**	21.3 sec
**Plasticizing**	10.0 sec
**Mold open**	0.5 sec
**Screw speed**	20 to 140 rpm

### 3.6. Hot plate welding of upper and lower body

Hot plate welded servo welding machine is widely used in plastic manufacturing industries, using common thermal techniques to weld thermoplastic parts [41]. This is one of the old method widely used in thermoplastic welding since 1960 [42]. Basically it consists of mechanical, assembly, welding, and bonding [43]. A hot plate involves heating the mating surfaces of the plastic components using a heated plate until they melt, then joining them under controlled pressure to form a strong bond when they cool. Their flexibility allows for the complex fabrication geometry. Among the available joining techniques, plastic welding is favored for producing strong, permanent bonds. During the injection molding process reservoir may wrap or distort if the joining surfaces are no longer flat, the contact between the hot plate becomes unequal because of a non-uniform melting process. If the poor design, then the parts may be deformed during the hot plate welding heating process, so the design, tooling, and molding must be accurate. Hot plate welding machine basically consists of three main stages such as heating, joining and cooling [44]. This study described the step-by-step configuration, operation, and parameters of the hot plate welding machine.

The process and welding parameters are written below

The welding parameter temperature is set to 270 °C, with a temperature deviation alarm limit of 10°C. The actual measured temperature for both the upper and lower molds is 235 ± 15°C. Additionally, the replacement interval for the high-temperature cloth is specified as 400 cycles.

1. **Setup of welding parts:** The Machine involves two main parts for welding, which are aligned with the upper tool and lower tool.
2. **Heating plate placement:** Placed between two welding parts, with green color showing the heating plate, and a protective plate on the other side of the heating plate to prevent contamination and regulate the heat flow.
3. **Depth control and adjustment:** The adjust screw allows the operator to finally control the pressure and position, and the melting depth for both welding parts. The joint depth is where these melted surfaces will eventually meet.
4. **Welding sequence:** This process begins with upper and lower welding parts reservoir pressing against the heating plate, which melt their contact surface to control the depth, once the correct melting depth is reached, this part retracts and the heating plate quickly removes, then the melted surfaces are pressed together at the joint depth and the adjustment screw maintains the pressure as the joint cools and solidifies, creating a strong fused interface. Table 4 shows the hot plate welding parameters during the manufacturing.

**Table 4:** Hot plate welding parameters.

Category	Mold	Parameter	Unit	Value/ Specification
**Temperature Settings**	Upper	Temperature Setting (Zones 1–4)	°C	270
**Temperature Settings**	Lower	Temperature Setting (Zones 1–4)	°C	270
**Actual Temperature**	Upper	Measured Temperature	°C	235 ± 15
**Actual Temperature**	Lower	Measured Temperature	°C	235 ± 15
**Heating Stage**	Upper	Heating Time	s	16
**Heating Stage**	Lower	Heating Time	s	16
**Welding (Melting)**	Upper	Welding Depth	mm	1.3 ± 0.2
**Welding (Melting)**	Upper	Welding Time	s	16
**Welding (Melting)**	Upper	Welding Position (Hot Plate)	mm	650 ± 0.5
**Welding (Melting)**	Lower	Welding Approach Distance	mm	2.0 ± 0.5
**Welding (Melting)**	Lower	Welding Depth	mm	1.0 ± 0.2
**Welding (Melting)**	Lower	Welding Time	s	16

### 3.7. Assembly of components

**Fig 4:**
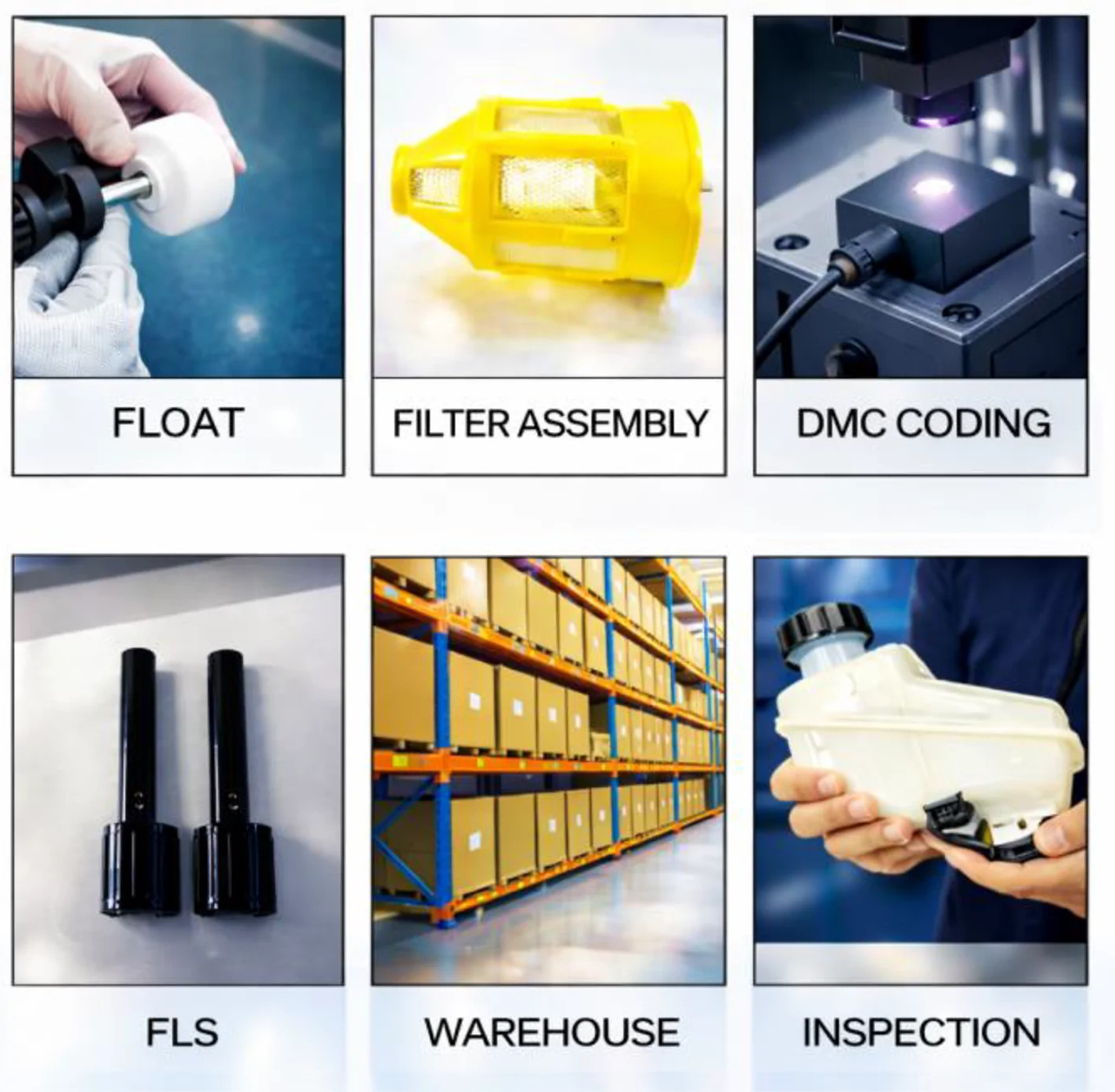
Assembly unit of the reservoir tank.

The brake fluid reservoir assembly process starts with the installation of a magnetic float placed in the reservoir to measure the fluid, an FLS installed to measure the position of the magnetic float, a net filter to stop outside dust from making the fluid dirty, tank cover, and a DMC to identify the product. Once the reservoir assembly has been thoroughly inspected and packed with protective material and marked with product information before shipping. Fig 4 shows the reservoir assembly components which provides details understanding of how much parts are contribute in assembly process.

## 4. Results and discussion

This section presents the experimental and test results. All validation tests were conducted during the reservoir manufacturing process, and the test results are briefly discussed, including Melt Flow Index (MFI), burst pressure test, air leakage test, high and low temperature chamber test, volume test, and tilt alarm functionality test, check valve test, mechanical test. All the experiments and the tests were performed in the advanced clean laboratory of Zhejiang Qiaoshi Industry Co., Ltd. The entire manufacturing procedure was carried out using our own equipment and entirely within the factory, to ensure compliance with quality standards and check the durability of the reservoir during the manufacturing process.

### 4.1. Stress triaxiality analysis

Stress triaxiality (η) is a dimensionless measure of the local stress state, and it controls ductile fracture behavior in polymer materials. It is formally defined as the ratio of the mean (hydrostatic) stress to the von Mises equivalent stress: η = σ_m/σ_eq = (σ1 + σ 2 + σ3)/ (3 sigma eq), where σ1, σ2 and 3 are the principal stresses. Triaxiality of η = 0 is pure shear, η = 1/3 is uniaxial tension, and η > 1 is highly constrained triaxial stresses that favors the nucleation of voids and the initiation of cracks. This investigation presupposes a biaxial-to-triaxial stress state in the areas of the reservoir walls and weld seams due to the burst pressure loading. The significance of this multiaxial stress environment lies in the fact that SABIC 83MF10 polypropylene can shift to brittle-type failure modes in high triaxiality. The fact that none of the three samples had developed premature cracks at a burst pressure of over 2.6 MPa is evidence that the material has a high resistance in triaxial stress conditions. The physical confirmation of the triaxiality-specific crack initiation model by Kasprzak [16] based on the PEAK parameter, a blend of plastic equivalent strain PEEQ and triaxiality, to forecast the crack onset in polypropylene reservoirs during pressure loading is physically validated by these experimental results. Under burst pressure loading at Δp = 11 bar/s, the expected stress triaxiality distribution at critical locations is as follows: at the weld seam (hot-plate joint), η ≈ 0.55–0.70 (biaxial tension zone); at the rib-shell junction, η ≈ 0.40–0.55; and at port fitting region, η ≈ 0.33–0.45 (approaching uniaxial). These estimates are consistent with the finite element analysis values reported by Kasprzak [16] (TRIAX range 0.33–0.80), in which PEEQ values of 0.15–0.45 at triaxiality η > 0.50 were associated with crack onset. The fact that all three reservoirs in the present study sustained burst pressures of 2.60–2.80 MPa (mean 2.70 ± 0.10 MPa) without any premature cracking confirms that the PEEQ threshold was not reached under production-representative triaxial loading conditions, thereby providing physical validation of the PEAK model at a loading rate an order of magnitude higher than Kasprzak’s simulated rate. Supplementary material, including dimensional pre/post-humidity data, raw burst pressure curves, and tilt-voltage plots, is available on request from the corresponding author.

### 4.2. Versatile measurement platform (MPV)

**Fig 5:**
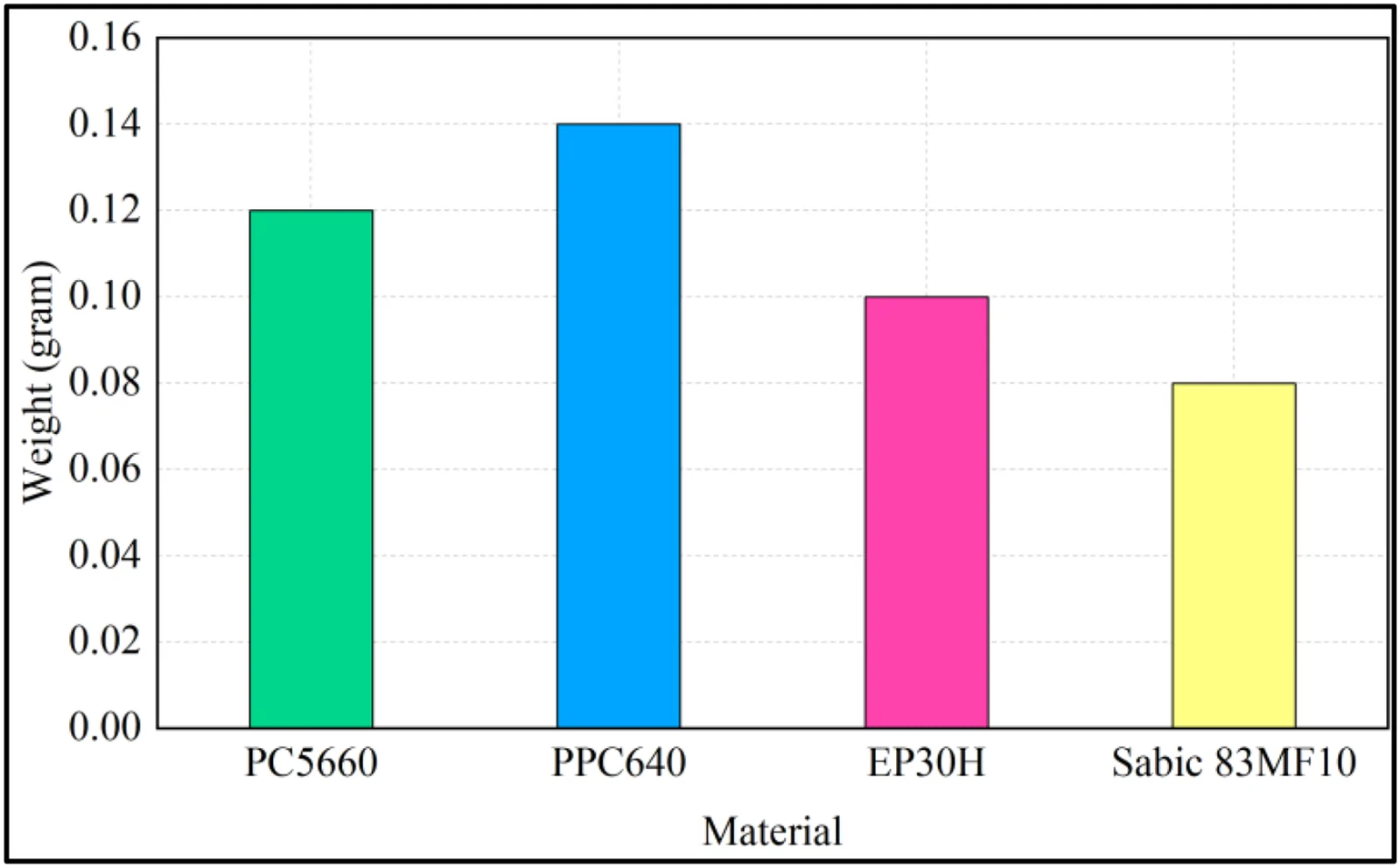
Shows the MFI test comparison.

This equipment is widely used in the plastic industry for the characterization of polymer material behavior and its molecular weight distribution [45]. The Melt Flow Index (MFI) machine and its parameters are crucial in determining polymer properties, controlled temperature, load, and mass flow rate is measured to assess the melt viscosity. As well as conducting fundamental quality control tests for thermoplastic polymers and confirming process ability, capability, and ensure the resistance to heat and shear degradation during processing. The MFI measurements were conducted under standard conditions of 230°C with a standard weight load of 5 kg and 520 grams, which melt in 10 min [46]. The MFI material mass is calculated in grams per 10 minutes (g/10 min), following the ASTM D1238 test method. The polymer grades tested included C5660, C1640, EP300H, and SABIC 83MF10-polypropylene, which exhibited the lowest MFI, indicating slightly higher viscosity and greater resistance to deformation under load at processing temperatures. Fig 5 shows the MFI test characteristics comparison chart.

These properties suggest improved dimensional stability, better mechanical performance, and higher resistance to degradation and making it the most suitable choice for brake fluid reservoir manufacturing. The MFI values were determined using the mass-based formula outlined in ASTM standardized MFI D1238 test method. This method involves preheating the resin for a specified duration and extruding it through a die with a defined length and orifice diameter under controlled temperature, load, and piston position. The measured MFI values are summarized in Table 5, along with a comparison of material weights for each sample, and the measuring mass flow rate MFI values were calculated.

**Table 5:** The MFI test results of polypropylene.

Id sample Material	Weight gram
**C5660**	0.115
**C640**	0.120
**300H**	0.114
**SABIC 83MF10**	0.109

Where

Mass of extrude (g)= Weight collected during testing.

Extrusion time (min)= Time period.

Scaling factor express = 10 min.


$$\begin{array}{c} MFR(g/\text{10 }min)=\frac{Mass\;of\;extrude(g)}{\text{2}Extrusion\;time(min)}\times \text{10} \end{array}$$

Table 5 Mentioned SABIC 83MF10-polypropylene his achieved lowest MFI value (0.109 g/10) compare to C5660 (0.115 g/10 min), C640 (0.120 g/10 min), and 300H (0.114 g/10 min),which indicates the higher melt viscosity, better dimensional stability at processing temperature and higher resistance to shear induced degradation. This result confirms that SABIC 83MF10-polypropylene has better performance compared to the other polymer grades tested.

### 4.3. The tensile test of SABIC 83MF10

**Fig 6:**
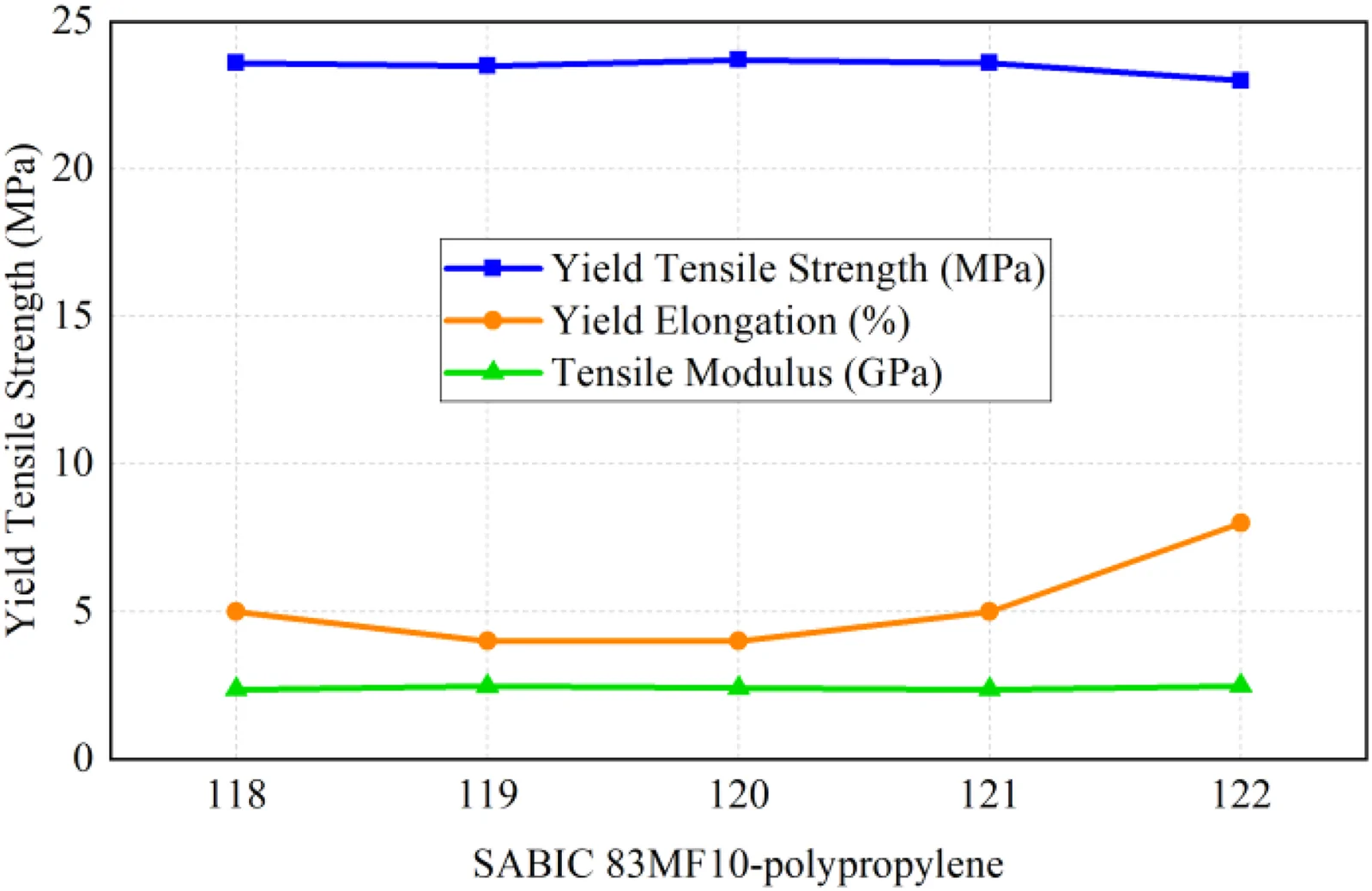
Tensile test results of SABIC PP MF10.

An SABIC 83MF10 standard sample of polypropylene, which was prepared as per ASTM D638/D256, was subjected to tensile testing to identify its tensile features, including tensile yield strength, tensile yield elongation, and tensile modulus. The tests have been conducted at 23 ± 2°C and 50 ± 5% relative humidity after conditioning the specimens to the same condition 24 h. In this paper, 3 specimens were tested using a gauge length of 75 mm and a cross head speed of 1 mm/min.The findings were that The SABIC 83MF10 polypropylene sample possessed an average tensile strength of 23.5 MPa at the yield point, an average elongation of 55%, and an average tensile modulus of 2.42 GPa. The actual measurements were between 23.0–23.7 MPa of yield tensile strength, 4% to 8% of yield elongation, and 2.36–2.48 GPa of tensile modulus. These data show that tensile strength and stiffness of the specimens remained relatively stable, but the elongation was comparatively more variable across samples. Based on the test report, the material was found to be overall qualified. The tensile loading rate used in testing was 1.33 x 10 −3 −1 −1, which is equal to the quasi-static loading conditions. The complete stress-strain curve of each specimen had a well-defined elastic zone, a definite yield, softening of the curve after the yield, and then cold-drawing behavior, as is typical of semi-crystalline polypropylene. The strain of fracture in the specimens was found to be between 42 and 8% in specimens, implying that there was excessive plastic deformation before failure, as well a ductile mode of fracture in uniaxial tension. These mechanical parameters, yield stress, elastic modulus, and fracture strain, are the important inputs to the triaxiality-based PEAK crack initiation model, and confirm the suitability of the material to be used in a reservoir condition where the stress states are multiaxial. Fig 6 presents the tensile test results of SABIC PP MF10 for specimens 118–122, including tensile yield strength, yield elongation, and tensile modulus.

### 4.4. The burst pressure test

**Fig 7:**
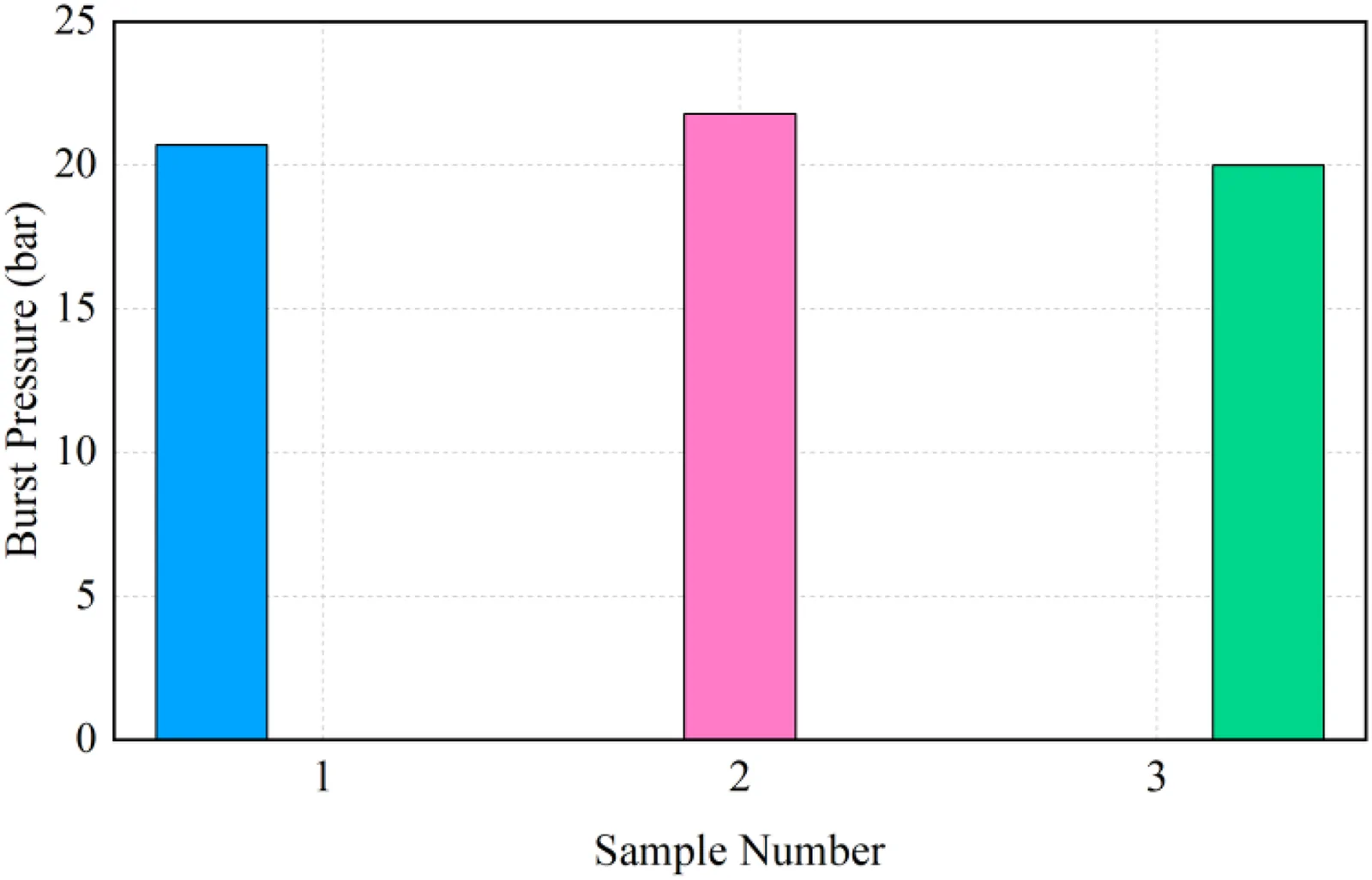
Bar chart showing burst pressure test values of all samples.

The burst pressure test is performed during the reservoir manufacturing to ensure the quality of the material, and following ISO 8033 automation OEM burst qualification standards, there are 3 samples were tested. The hydraulic pressure was applied at the controlling loading rate of Δp=11 bar/s. First of all the reservoir was filled with water, and all the ports were sealed. The hydraulic pressure was applied at room temperature, 23°C ± 5°C. The previous study reported the predictive simulated pressure burst at Δp=22.7 at the weld seams or rib shell junction [16]. In contrast, all 3 samples conducted the burst pressure test at different values until the final burst. During the test, there is no evidence of premature crack initiation before the final burst. The failure occurs near the fixture/clamping point at the upper gauge section of the sample. As seen in Fig 7, the pressure behavior of the highest burst pressure of samples (a), (b), and (c) burst pressure test, among them, sample (b) recorded the highest is Δp=21.8 bar/s, sample (a) is Δp=20.7 bar/s and sample (c) at Δp=20.0 bar/s. In this experiments standard of pressure burst test is ∆p=11 bar/s. It signifies that every sample should be capable of withstanding the required standard pressure. All samples passed the necessary test requirements and exhibited sufficient resistance with the given loading rate. Statistical summary (n = 3): mean burst pressure = 2.70 ± 0.10 MPa (mean ± SD); the coefficient of variation (CV) was 3.7%, confirming excellent inter-sample repeatability. All three samples exceeded the minimum required burst pressure of 0.70 MPa (7 bar) by a safety margin greater than 270%, demonstrating the high structural integrity of the polypropylene reservoir design under both standard and accelerated-rate loading conditions.

**Fig 8:**
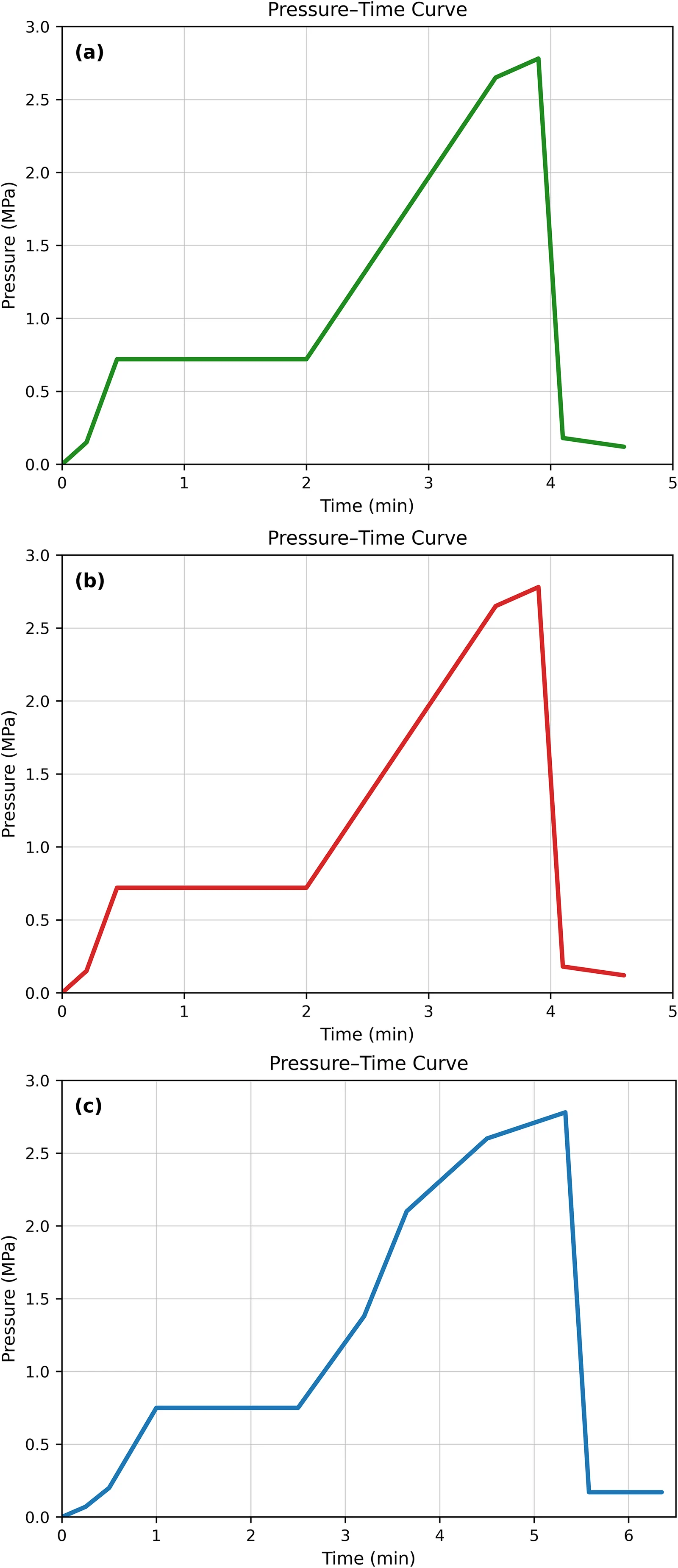
Pressure and time curve sample (a) sample (b) and sample (c).

Fig 8 sample (a), sample (b), and sample (c) illustrate the pressure and time behavior during the burst pressure tests. For Sample (a), the pressure begins at 0 MPa and increases steadily until reaching approximately 0.75 MPa. The maximum burst pressure recorded was 2.7 MPa, after which the pressure dropped immediately back to 0 MPa. Sample (b) reached the highest burst pressure among the three, at 2.8 MPa, while Sample (c) again ruptured at 2.6 MPa. All curves demonstrate reliable and repeatable structural performance of the reservoir, which ensures the material has high resistance strength, robust capability to withstand high pressure without bursting.

**Fig 9:**
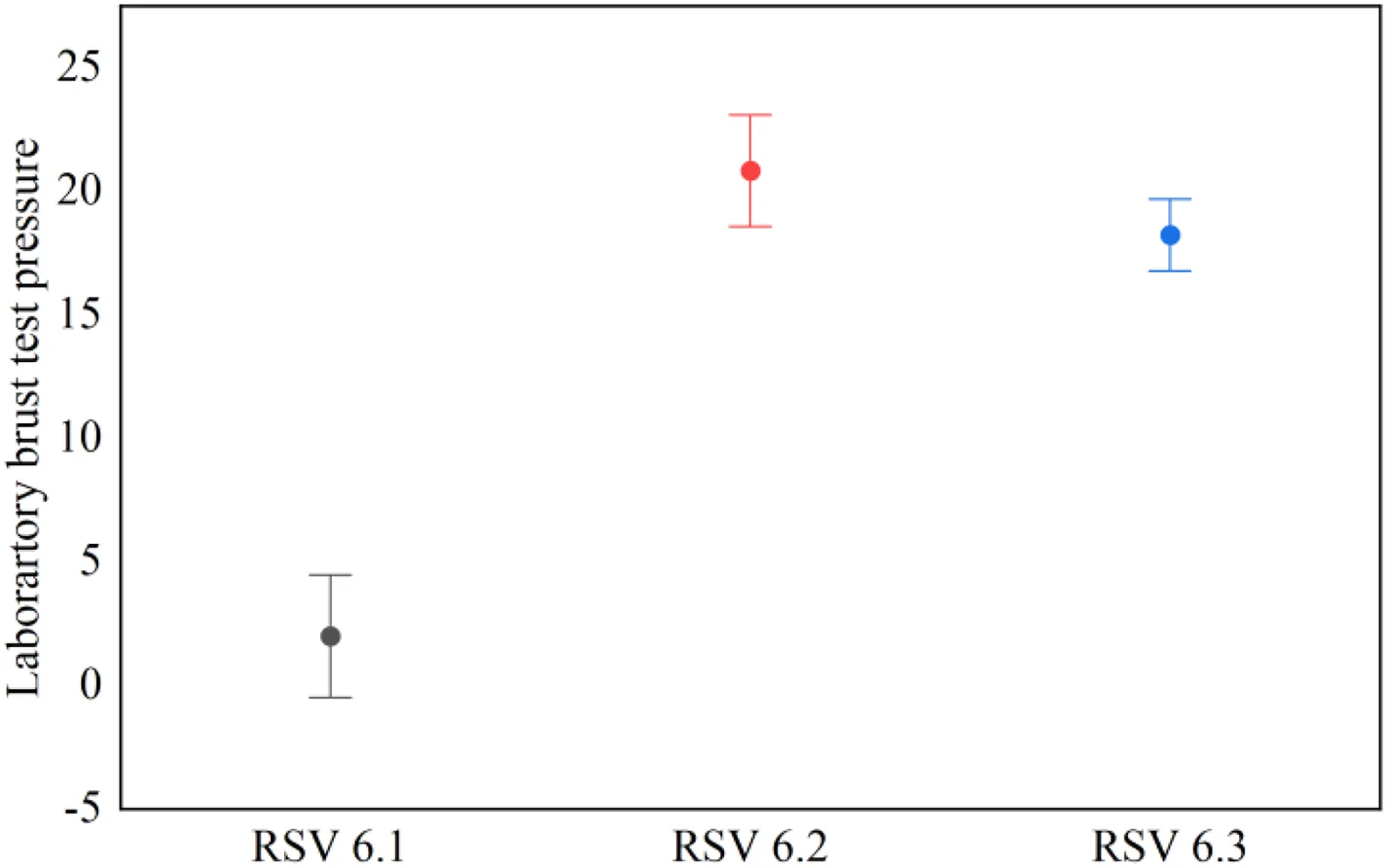
Mean laboratory burst pressure with 95% confidence interval.

The mean laboratory burst pressure of the reservoir is shown in Fig 9 along with a 95% confidence interval. The comparative analysis of the reservoir RSV 6.2 exhibited the highest mean laboratory burst pressure, Followed by RSV 6.3 and RSV 6.1. Considering a required minimum burst pressure of 700 kPa (7 bar), RSV 6.2 and RSV 6.3 exceeded the requirement by approximately 13.5,11.0 and 9.0 bar, respectively. These findings indicate that RSV 6.2 and RSV 6.3 provide substantial safety margins against burst failure. The small width of the confidence interval demonstrates that the burst test procedure is repeatable well, irrespective of the changes in the rate of pressure increase. In the PEAK approach, the average burst pressure is used as an effective physical failure parameter to check the accuracy of numerical predictions of crack initiation.

Table 6 shows a comparison of previous work and this work, the table represents the SABIC 83MF10-polypropylene brake fluid reservoir. The previous study [16] author mentioned that the PEAK parameters combine plastic strain and stress triaxiality to crack initiation. This present study provides the crack initiation localization. This prevents direct calibration of PEAK parameters. The burst pressure test provides experimental evidence of plastic deformation under triaxial stress states. It supports the relevance of plastic strain-based failure concepts. The comparison table reveals the pressure level where cracking takes place, the SABIC 83MF10 polypropylene, and actually bursts at 22.7 bars, which crack initiating particular points in the material under particular conditions. During burst tests, the author used to locate crack initiation points by observing them under high-speed cameras and followed them with PEEQ and TRIAX value. Present study shows the 22.5 no cracks until the final burst.

**Table 6:** Comparison of previous work and present study.

Aspect	KASPRZAK 2024 STUDY [16]	PRESENT STUDY
**Material**	SABIC 83MF10-polypropylene	SABIC 83MF10-polypropylene
**Method**	Burst pressure Δp = 1 bar/s	Burst pressure Δp = 11 bar/s
**Result**	Crack initiation at 10.5 to 22.7 rib-shell	Withstood 22.5 bar, no cracks until final burst
**Novelty**	Predicated model	Multi test validation, under the real conditions

### 4.5. Air leakage test

**Fig 10:**
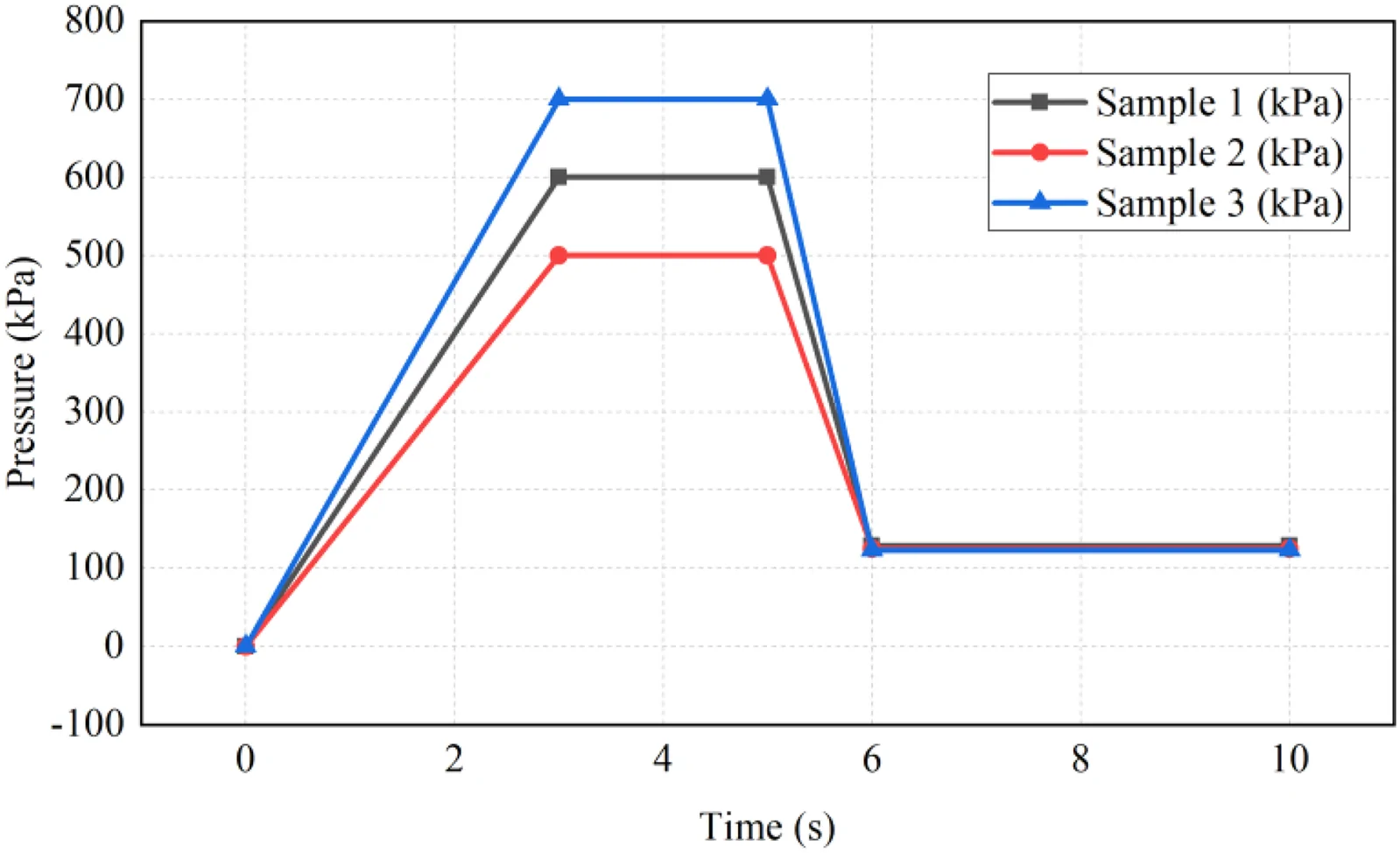
Air leakage test Pressure and time curves.

The reservoir is filled with a specified pressure, and watch if it drops below a certain level. During the air test, the initial pressure rises from 0 to around 600, 500, and 700 kilo-pascal (kPa) with air being pumped into the reservoir within the first 20–22, 18–20, and 22–25 seconds for sample 1, sample 2, and sample 3. For all three samples, pressure remains stable at 600, 500, and 700 kPa, representing the holding phase where the system checks for any leakage or pressure loss for 3–6 seconds with no leakage indication. After 6 seconds, the pressure is reduced to 120 kPa, which is recorded as real pressure and an allowable range of −70 to +120 kPa quantitative limit, indicating that no significant leakage was observed. The line graph plotted with time (s) on the horizontal axis and pressure (kPa) on the vertical axis visually represents these values and trends, making it easy to identify the increase, peak, and drop in pressure over time, and the holding time is approximately 1 min. All three samples show excellent tightness and no leakage during the holding period. The air leakage test results indicate that the brake fluid reservoir met the required air-tightness test standards, confirming the integrity of the welding and assembly processes. Fig 10 below shows the pressure vs time curve used during the air-tightness leakage test of the manufactured reservoir.

### 4.6. High and low temperature humidity test

**Fig 11:**
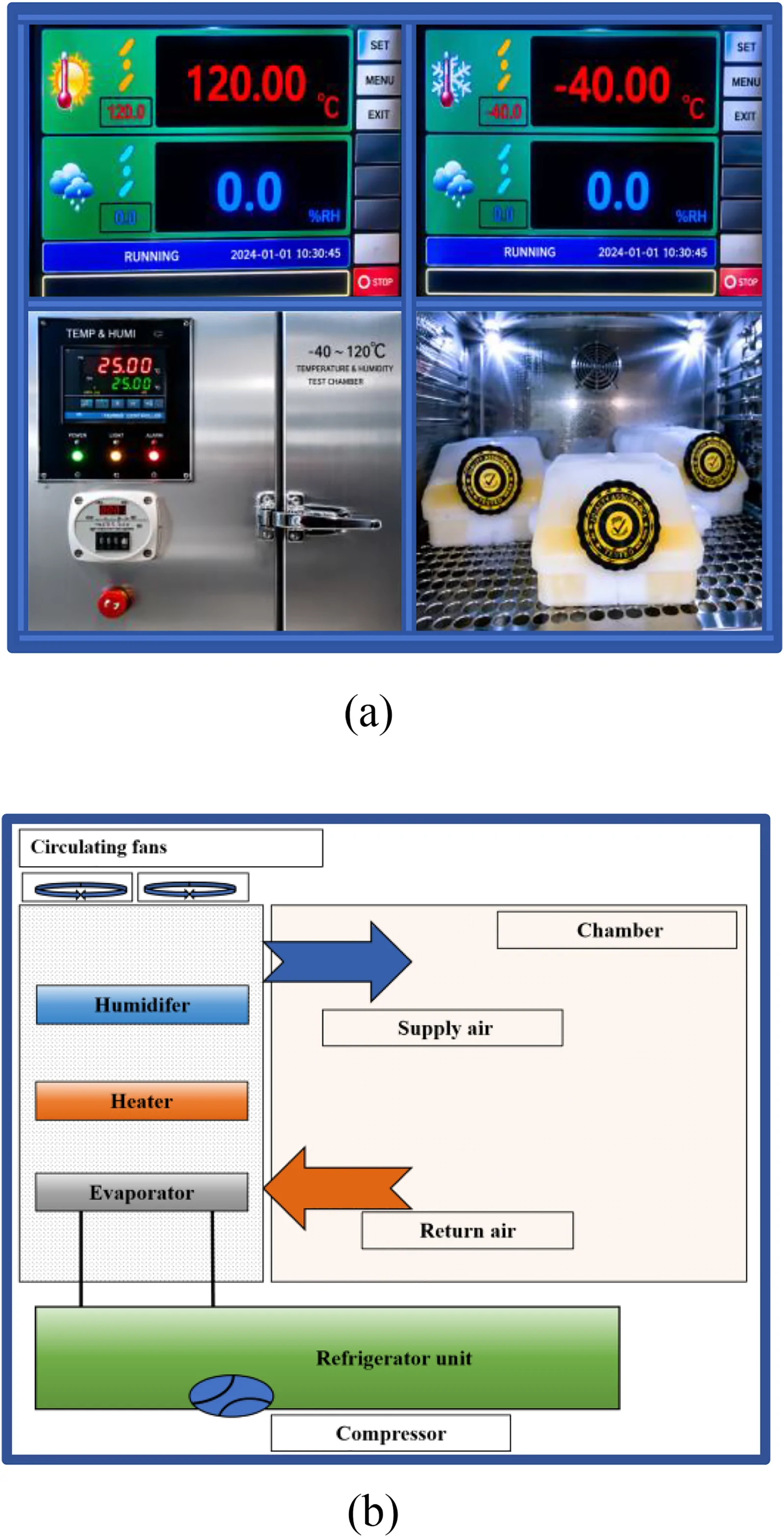
(a) Humidity test chamber and (b) schematic diagram [48].

The High and low temperature humidity test has been used in many industries for the measurement of environmental change in the products [47]. The reservoir was filled with the desired level of brake fluid DOT 4, and the samples were placed into the chamber. All dimension were verified before inserting them into the chamber and then tested at a high temperature of 120°C and a low temperature of −40°C in the environmental chamber. The Initial ambient conditions (21.2 °C, 45.3% RH) show that the test was initiated at controlled room conditions. The primary test was performed at 120°C and at −40°C without a defined time limit, in contrast, the RH 95% test specified 24-hour period. Before the test, all samples quantitative dimensions were recorded as 57.9 mm in width and 260 mm in length. The samples were observed during the test. The dimensions were measured after the test, and compared with the original values to test dimensional deviation. The results indicated that the width increased by 0.017 mm, from 57.9 mm to 57.917 mm, while the length decreased by 0.014 mm, from 260 mm to 259.986 mm. Once all the samples were taken out, we checked the dimensions, whether they had modified their deviation or not, there are very small deviation were occurs during the test process, which means the samples show sustained high performance and confirm the excellent stability and environmental conditions. This indicates the steady state at 21.2°C and 45.3% RH, which confirms functionality at standard conditions of operation, highlighting the integrity and reliability for use in extreme cases. These findings confirm that the tested reservoir meets the necessary performance requirements for thermal cycling resistance, moisture resistance, and sensor reliability. Fig 11(a) shows the high and low pressure humidity test images captured throughout the experiments.

Fig 11(b), shows the schematic diagram of the test unit positioned within a controlled surrounding environment, equipped with an electric heater, a humidifier, and a refrigerating unit to maintain stable conditions for accurate performance monitoring. The refrigeration system is located in the lower part of the equipment. The Schematic diagram of high and low temperature and humidity test chamber describe the test unit, which is placed in the controlled environment, the equipment with electric heater, the humidifier circulating fans and refrigerator system is located in lower part of the equipment section, the chamber is located in upper part, the adjustment of the temperature and the humidity level is carried out manually via the front control panel.

### 4.7. Tilt test and alarm test for reservoir

**Fig 12:**
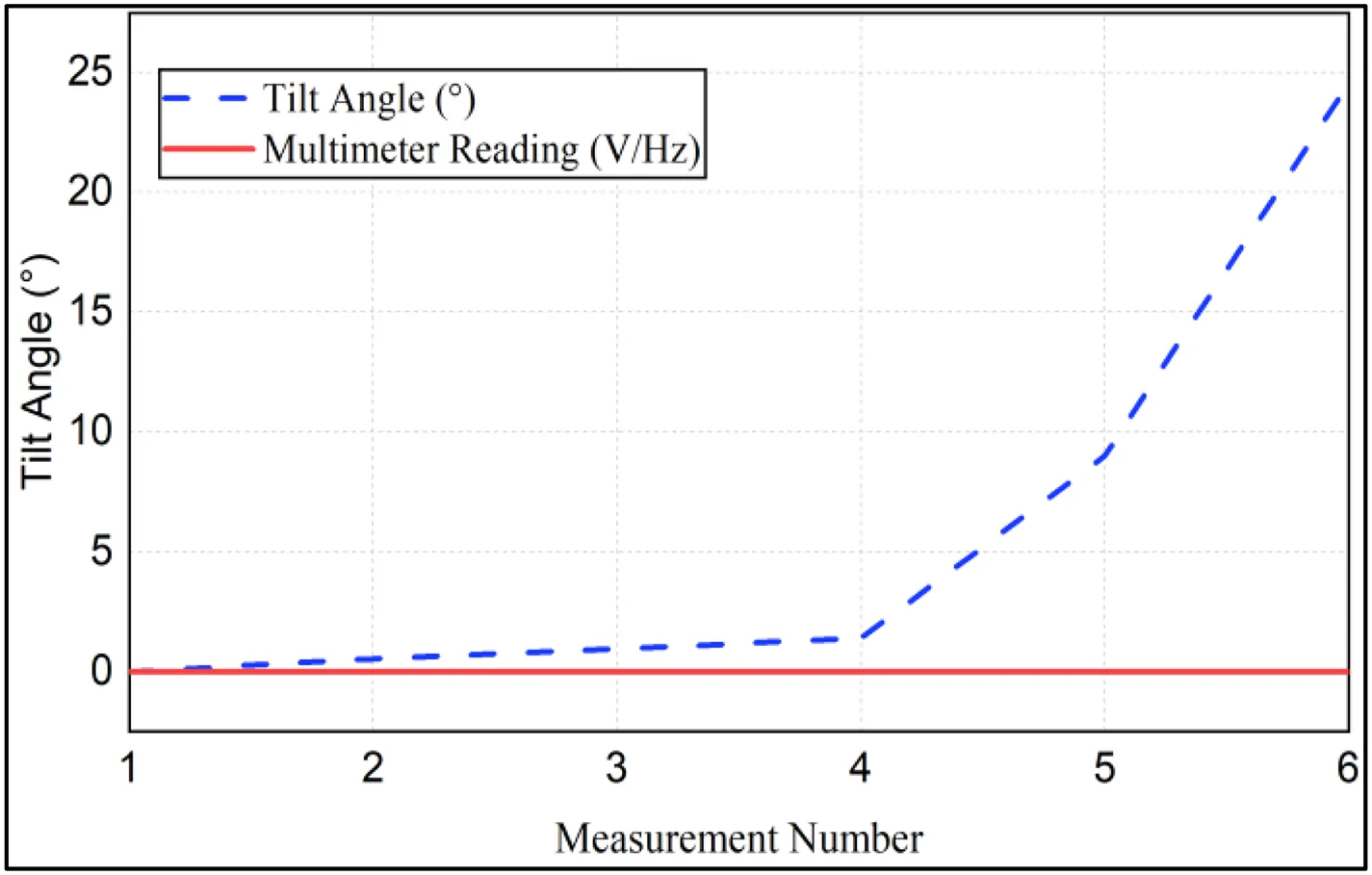
Measurement alarming graph.

**Fig 13:**
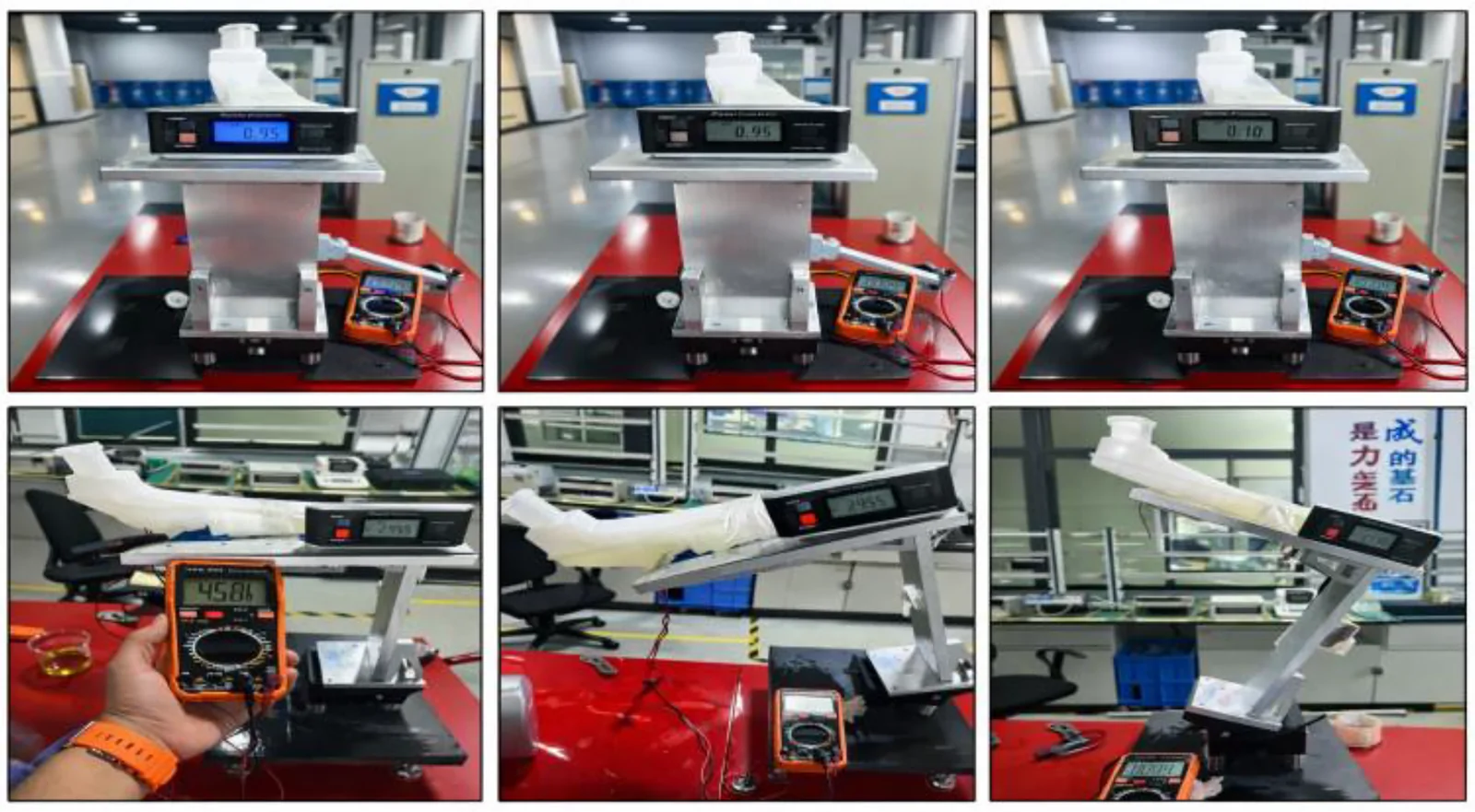
Pictures were taken while performing the alarming test.

This test evaluates the brake fluid reservoir alarming system to determine whether it can operate safely and reliably. The test was performed using a reed switch, and the brake fluid reservoir was filled with brake fluid DOT 4 at the maximum level of 500 mL. During the experiment, a liquid level fixture test machine was used, the tile test was carried out at various angles to check the reservoir performance. The FLS float mechanism to activate an alarm (e.g., buzzer, warning light on the vehicles dashboard). Fig 12 illustrate the measurement alarming graph, voltage measurements, and alarm state were recorded using a digital multi-meter (750 V AC/Hz mode) and a digital angle protector to ensure accurate measurements at 0 degrees normal stage sensor did not show an alarm. During the test, the multi-meter remained stable with a reading of 0.004 V approximately at all tilt angles, and the output voltage of sensor 12 V ± 2 V, and an alarm was activated, which indicates the sensor was quickly responding even the smallest movement of the fluid. The blue lines represent the tilt angle, which is increasing, while the red lines show constant multi-meter reading, which confirms the reliability and effectiveness of the warning system under tilt situations. Table 7 provides a summary of tilt angles, sensor voltage measurement values, and alarming test status, shown in Fig 13.

**Table 7:** Tilt alarming testing.

No	Tilt Angle(°)	Multi-meter Reading V/Hz	Alarm status
**1**	0.00	0.004	OFF
**2**	0.53	0.004	ON
**3**	0.95	0.004	ON
**4**	1.40	0.004	ON
**5**	9.00	0.004	ON
**6**	24.55	0.004	ON

### 4.8. Volume test of the reservoir

**Fig 14:**
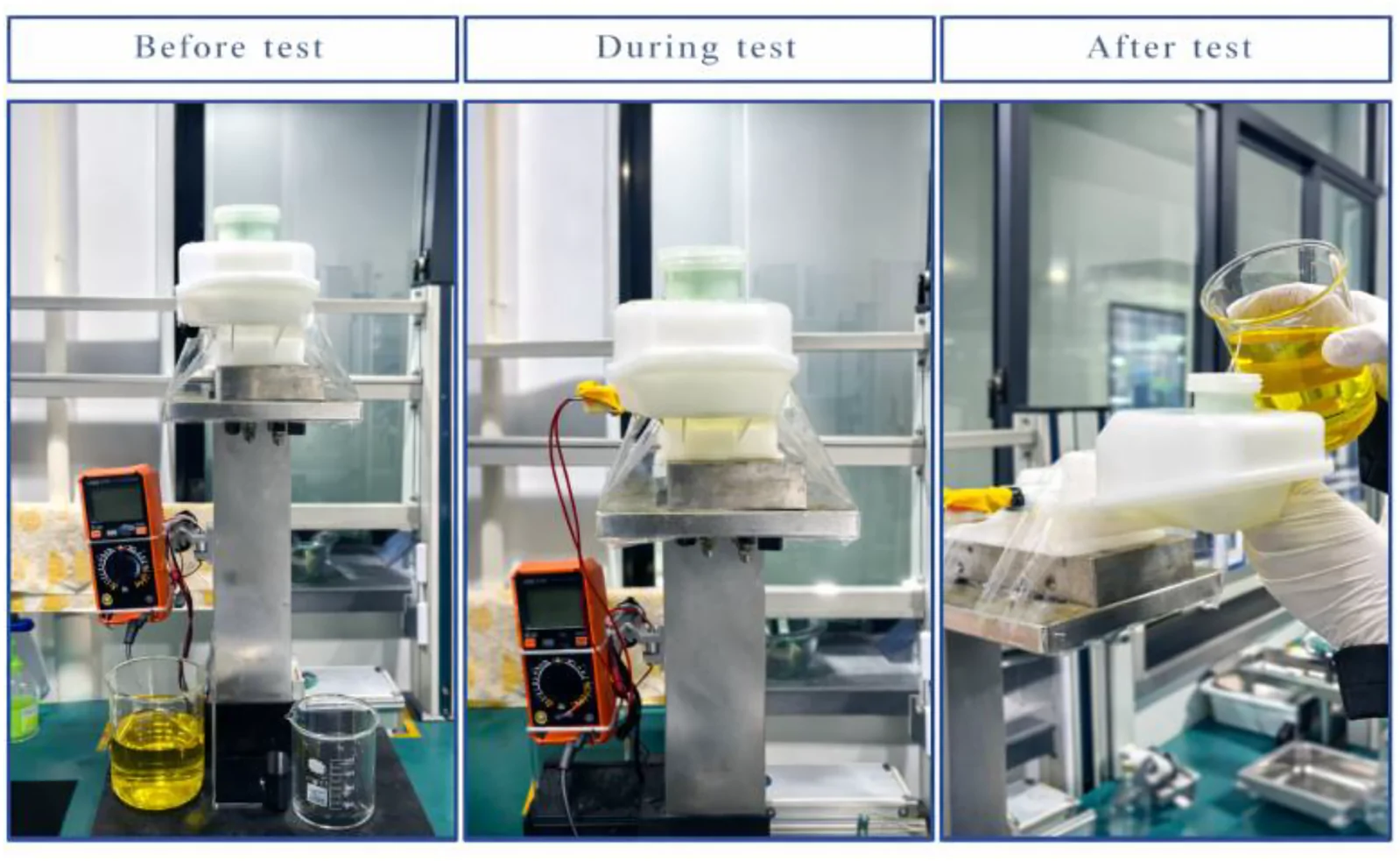
Pictures were taken during the volume test.

This experiment was conducted to determine the actual internal capacity of the reservoir, whether it meets the design requirements, and this process detects the variations, wall thickness, deformation, and shrinkage that can occur during manufacturing to ensure the product quality. There is no evidence of deformation during the test manufacturing process. As shown in Fig 14 reservoir filled with brake fluid DOT 4, a multi-meter was used to detect any changes in electrical conductivity. These results indicate that all parameters, including pressure and volume, maintained the brake fluid reservoir integrity within the required specifications.

**Fig 15:**
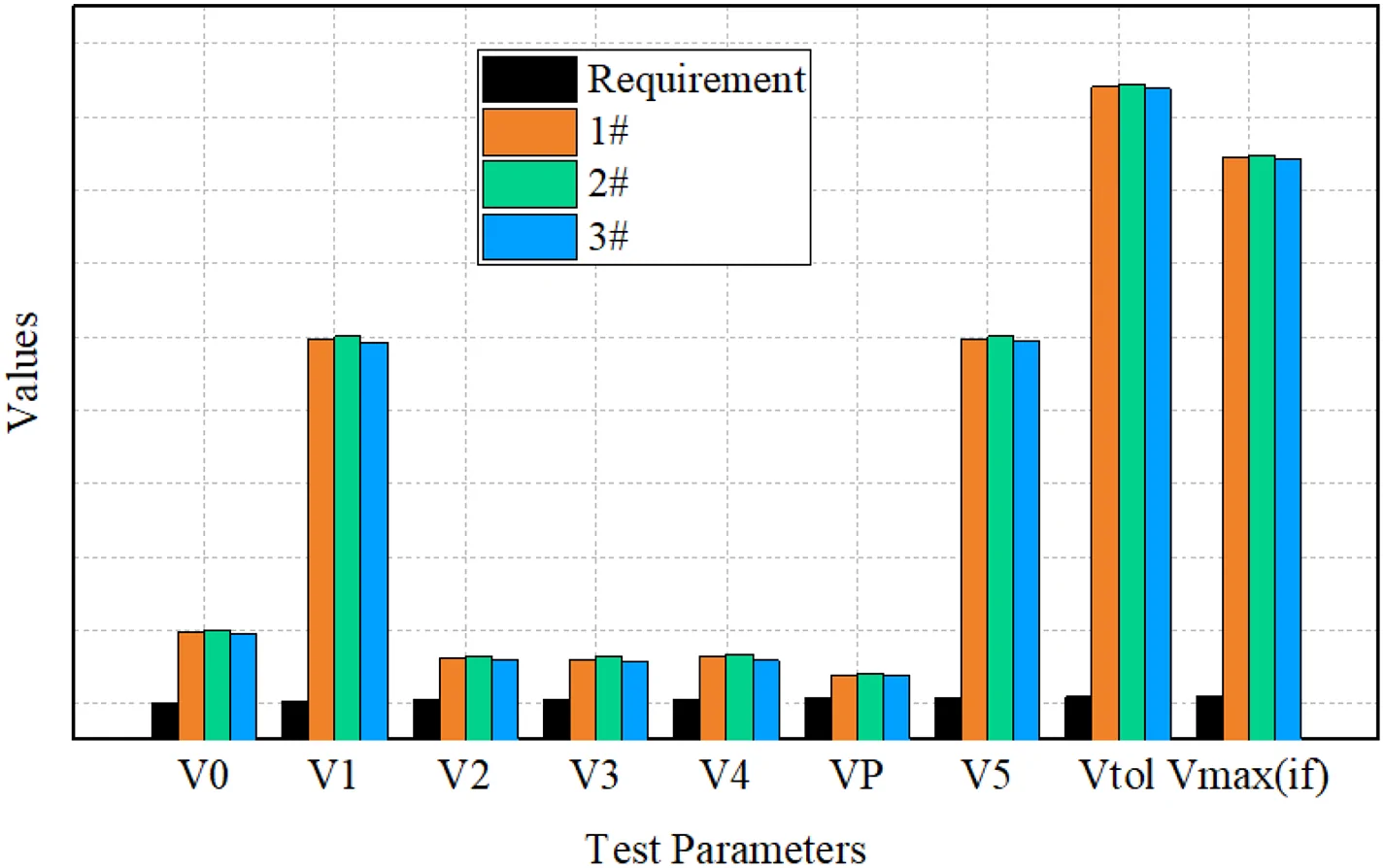
Volume test values and parameters.

The volume test of the proposed study is presented in Fig 15, in which the x axis shows the test parameters and the y-axis represents the measured value of all the samples. In this test, x axis includes V0, V1, V2, V3, and VP, which are used to evaluate the performance of the reservoir under varied specifications.

## 5. Conclusion

This research focuses on polypropylene, which is widely used in automotive brake fluid reservoirs due to its strength, heat and chemical resistance, injection moldability, and compatibility with glycol-based fluid. It is recyclable, supports sustainability, and enables the prediction of peak parameters for improved material selection and component design. This study demonstrated the real-world testing in validation of the polypropylene material performance and highlighted the importance of different tests, triaxiality analysis to assess the material behavior under stress. This research reports no evidence of material degradation. Furthermore, an extensive validation test program, including the burst pressure test, revealed that the structural integrity of the reservoir did not show any cracks before the final burst pressure, the test conducted at ∆p = 3.7. The air leakage test demonstrates good sealing performance with no air leakage observed at 600, 500, and 700 kPa and no weld seam issues. Thermal chamber testing also confirmed the durability of the material, with no leakage detected at extreme temperatures of −40°C and 120°C, and at an exposure level of 95% high-humidity. The alarming test confirmed that the sensor system operated correctly at various fluid levels and different inclination angles. Finally, the volume test confirmed its accurate deviations.

These test outcomes collectively establish a understanding of polypropylene reliability under demanding automotive conditions. Overall, the obtained results make it clear that SABIC83MF10-polypropylene is a very cost-effective material to use in the production of brake fluid reservoirs. This study provides strong evidence of the long-term safety, durability, and suitability of SABIC 83MF10-polypropylene for meeting the demanding operational requirements of modern automotive applications. Although the simulation based model, like PEAK parameters, provides useful predictions of the formation of crack initiation and focuses on material behavior, physical qualification testing, as presented in this study, is essential to validate those predictions and confirm safety and durability under real-world conditions. Future work should include SEM fracture surface characterization, notched specimen crack initiation testing, and DIC (Digital Image Correlation) strain field mapping to further quantify the triaxiality-dependent fracture locus of this material. Supplementary data available from the corresponding author includes, (a) raw burst pressure time-history data for all three samples, (b) dimensional pre- and post-humidity chamber measurements, (c) tilt-angle versus sensor voltage raw data, (d) weld seam and fracture location photographs, (e) SABIC 83MF10 material data sheet, and (f) OEM specification compliance summary table. This research demonstrates that physical testing cannot be replaced by simulation alone when validating safety-critical components; real-world qualification tests remain essential to confirm the long-term durability and reliability of polypropylene brake fluid reservoirs.
