# Supplementary material for: Investigation of crack initiation, and mechanical behavior of polypropylene and polymer-based composites under plastic deformation in automotive brake fluid reservoirs
Source: PLoS One. 2026 Sep 18;21(9):e0352763. doi: 10.1371/journal.pone.0352763 (PMC13588379; doi:10.1371/journal.pone.0352763)
Supplement: Supplementary file 1 [file pone.0352763.s003.pdf]

Journal Requirements:

When submitting your revision, we need you to address these additional requirements.

Comment 1. Please ensure that your manuscript meets PLOS ONE's style requirements, including those for file naming. The PLOS ONE style templates can be found at

https://journals.plos.org/plosone/s/file?id=wjVg/PLOSOne_formatting_sample_main_body.pdf and

https://journals.plos.org/plosone/s/file?id=ba62/PLOSOne_formatting_sample_title_authors_affiliations.pdf

Response 1. Thank you for your comment. We have carefully checked and revised the manuscript to meet PLOS ONE's style and formatting requirements, including file naming. The manuscript has been reformatted in Word according to the PLOS ONE templates provided, and all relevant submission files have been updated accordingly. All identified formatting issues have been corrected in the revised version.

Comment 2. Please update your submission to use the PLOS LaTeX template. The template and more information on our requirements for LaTeX submissions can be found at http://journals.plos.org/plosone/s/latex.

Response 2. Thank you for your comment. We understand the journal's requirement to use the PLOS LaTeX template. At present, the revised manuscript has been prepared in Word format following the PLOS style requirements. We would be grateful for the editor's guidance on whether Word format is acceptable for the revised submission, or whether conversion to the PLOS LaTeX template is mandatory at this stage.

Comment 3. We note that the grant information you provided in the 'Funding Information' and 'Financial Disclosure' sections do not match. When you resubmit, please ensure that you provide the correct grant numbers for the awards you received for your study in the 'Funding Information' section.

Response 3. Thank you for pointing this out. The authors have no other funding for this manuscript.

Funding Information / Financial Disclosure:

The authors have no other funding for this manuscript.

Author Contributions

Conceptualization: JH.

Data curation: LJ JH.

Formal analysis: KW JH.

Investigation: AR MH LJ.

Methodology: AR JH.

Project administration: JH ZD.

Resources: JH ZS.

Software: MH YQ.

Supervision: KW.

Validation: ZD YB.

Visualization: ZS.

Writing – original draft: JH JL.

Writing – review & editing: JH AR KW.

Comment 4. Thank you for stating the following in the Financial Disclosure section:

"Zhejiang Qiaoshi Industry Co., Ltd. pay funding"

We note that one or more of the authors are employed by a commercial company:

- Zhejiang Qiaoshi Industry Co., Ltd.,
- Ningbo Qiaoshi Rubber & Plastics Co., Ltd.,

1. Please provide an amended Funding Statement declaring this commercial affiliation, as well as a statement regarding the Role of Funders in your study. If the funding organization did not play a role in the study design, data collection and analysis, decision to publish, or preparation of the manuscript and only provided financial support in the form of authors' salaries and/or research materials, please review your statements relating to the author contributions, and ensure you have specifically and accurately indicated the role(s) that these authors had in your study. You can update author roles in the Author Contributions section of the online submission form.

Please also include the following statement within your amended Funding Statement.

“The funder provided support in the form of salaries for authors [insert relevant initials], but did not have any additional role in the study design, data collection and analysis, decision to publish, or preparation of the manuscript. The specific roles of these authors are articulated in the ‘author contributions’ section.”

If your commercial affiliation did play a role in your study, please state and explain this role within your updated Funding Statement.

2. Please also provide an updated Competing Interests Statement declaring this commercial affiliation along with any other relevant declarations relating to employment, consultancy, patents, products in development, or marketed products, etc.

Within your Competing Interests Statement, please confirm that this commercial affiliation does not alter your adherence to all PLOS ONE policies on sharing data and materials by including the following statement: "This does not alter our adherence to PLOS ONE policies on sharing data and materials.” (as detailed online in our guide for authors http://journals.plos.org/plosone/s/competing-interests) . If this adherence statement is not accurate and there are restrictions on sharing of data and/or materials, please state these. Please note that we cannot proceed with consideration of your article until this information has been declared.

Please include both an updated Funding Statement and Competing Interests Statement in your cover letter. We will change the online submission form on your behalf.

Response 4. Amended Funding Statement:

Thank you for your comment. The authors have no other funding for this manuscript.

Comment 5. Please provide a complete Data Availability Statement in the submission form, ensuring you include all necessary access information or a reason for why you are unable to make your data freely accessible. If your research concerns only data provided within your submission, please write "All data are in the manuscript and/or supporting information files" as your Data Availability Statement.

Response 5. Thank you for your valuable comment. The data Availability Statement has been included in the revised manuscript and submission form. Data availability statement: All relevant data are included within the manuscript file

.

Comment 6. Please amend the manuscript submission data (via Edit Submission) to include authors:

Liang Jian Chun

Ying Quanjia

Yang Bin

Zhang Debing

Zhou Sen

Kefu Wang

Asif Raza

Malik Haris

Jibran Hussain

Response 6. Thank you for bringing this to our attention. We have revised the manuscript submission data via Edit Submission and added the requested authors. The author information has been updated accordingly in the submission system.

We have amended the manuscript submission data via Edit Submission to include all authors listed in the manuscript. We have also ensured that the author names, affiliations, funding statement, competing interests statement, and author contributions are consistent between the manuscript file and the online submission system.

Liang Jianchun 1, Ying Quanjia 1, Yang Bin 1, Zhang Debing 1,2, Zhou Sen 1,2, Wang Kefu 1,2, Asif Raza 4, Malik Haris 4, Jibran Hussain 1,3*

1. Ningbo Qiaoshi Rubber & Plastic Co., Ltd., Ningbo 315470, China
2. Zhejiang Qiaoshi Industry Co., Ltd., Ningbo 315470, China
3. School of Control Science and Engineering, Tianjin University, Tianjin 300072, China
4. School of Information Science and Engineering, NingboTech University, Ningbo 315100, China

Comment 7. Please amend either the abstract on the online submission form (via Edit Submission) or the abstract in the manuscript so that they are identical.

Response 7. Thank you. The abstract has been corrected so that the version in the online submission system matches the version in the manuscript exactly.

Abstract

This paper discusses the intensive research on polypropylene, a significant polymeric material, and its extensive application in the automotive industry, particularly in the manufacture of brake fluid reservoirs through a series of laboratory destruction tests and experiments, The paper emphasizes the triaxiality (Triax) ($\eta = \sigma m/\sigma eq$) is used as the theoretical framework for evaluating crack initiation and behavior under various stress states and critical factors for the understanding of crack formation intention and the mechanical behavior of polypropylene under mechanical loads. This study lies in the detailed evaluation of polypropylene behavior under various manufacturing conditions, addressing potential deviations that could affect final product quality. The findings demonstrate the material's robustness, and its high fracture toughness, thermal stability, and mechanical strength minimize crack initiation, leakage, damage, and other defects during the manufacturing process, ensuring reliable use in reservoir

production. The research methodology involved a series of tests and experimental procedures, such as (i) Melt Flow Index (MFI = 0.109 g/10 min, ASTM D1238, 230°C/5 kg); (ii) uniaxial tensile testing (yield strength: 23.0–23.7 MPa, tensile modulus: 2.36–2.48 GPa, fracture strain: 42–68%, ASTM D638, n = 3); (iii) burst pressure testing (mean burst: 2.70 ± 0.10 MPa at Δp = 11 bar/s, minimum requirement: 0.70 MPa, ISO 8033); (iv) air leakage testing (no leakage at 500–700 kPa hold for 60 s); (v) thermal chamber cycling (−40°C to 120°C, 95% RH, 24 h; dimensional change < 0.02 mm); (vi) tilt alarm test (alarm activation at ≤ 0.53° tilt); and (vii) volume capacity verification (within OEM specification) during manufacturing of the brake fluid reservoir. These results confirm the long-term suitability of SABIC 83MF10-polypropylene for safety-critical reservoir applications in modern automotive braking systems. The findings demonstrate the material's robustness, and its high fracture toughness, thermal stability, and mechanical strength minimize crack initiation, leakage, damage, and other defects during the manufacturing process, ensuring reliable use in reservoir production. The novel aspects of this study are the demonstration of the mechanical behaviour of polypropylene and the valuable insights provided into the brake fluid reservoir manufacturing process and performance, offering significant implications for automation in the automotive sector.

Key words; Polypropylene; Crack initiation; Plastic Strain; Material Behavior; Brake Fluid Reservoir; Automotive Materials.

Comment 8. Please ensure that you refer to Figure 12 in your text as, if accepted, production will need this reference to link the reader to the figure.

Response 8. Thank you for your comment. During revision, additional figures were inserted into the manuscript, and therefore the figure originally numbered as Figure 12 has now been renumbered as Figure 13. We have carefully checked the manuscript and ensured that the figure is appropriately referred to in the text under its revised numbering.

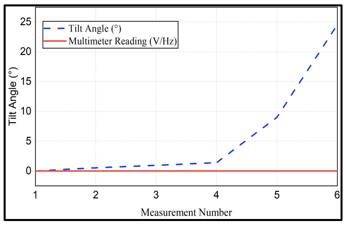

Figure 12 illustrate the measurement alarming graph

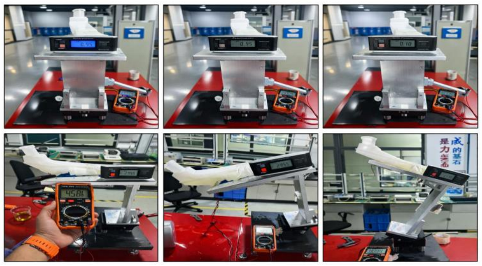

Fig. 13. Pictures were taken while performing the alarming test

Comment 9. We note that Figures 1, 4, 10, 12, and 13 in your submission contain copyrighted images. All PLOS content is published under the Creative Commons Attribution License (CC BY 4.0), which means that the manuscript, images, and Supporting Information files will be freely available online, and any third party is permitted to access, download, copy, distribute, and use these materials in any way, even commercially, with proper attribution. For more information, see our copyright guidelines: http://journals.plos.org/plosone/s/licenses-and-copyright.

We require you to either (1) present written permission from the copyright holder to publish these figures specifically under the CC BY 4.0 license, or (2) remove the figures from your submission:

1. You may seek permission from the original copyright holder of Figure 1, 4, 10, 12, and 13 to publish the content specifically under the CC BY 4.0 license.

We recommend that you contact the original copyright holder with the Content Permission Form (http://journals.plos.org/plosone/s/file?id=7c09/content-permission-form.pdf) and the following text:

“I request permission for the open-access journal PLOS ONE to publish XXX under the Creative Commons Attribution License (CCAL) CC BY 4.0 (http://creativecommons.org/licenses/by/4.0/). Please be aware that this license allows unrestricted use and distribution, even commercially, by third parties. Please reply and provide explicit written permission to publish XXX under a CC BY license and complete the attached form.” Please upload

the completed Content Permission Form or other proof of granted permissions as an "Other" file with your submission.

In the figure caption of the copyrighted figure, please include the following text: “Reprinted from [ref] under a CC BY license, with permission from [name of publisher], original copyright [original copyright year].”

2. If you are unable to obtain permission from the original copyright holder to publish these figures under the CC BY 4.0 license or if the copyright holder’s requirements are incompatible with the CC BY 4.0 license, please either i) remove the figure or ii) supply a replacement figure that complies with the CC BY 4.0 license. Please check copyright information on all replacement figures and update the figure caption with source information. If applicable, please specify in the figure caption text when a figure is similar but not identical to the original image and is therefore for illustrative purposes only.

Response 9. Thank you for your comment. We would like to clarify that the images included in Figures 1, 4, 10, 12, and 13 are primarily original photographs of our company’s machines, equipment, and experimental setup. Therefore, these images are owned by the authors and do not constitute copyrighted third-party material. Where any figure contains material adapted from previously published references, the original sources have been appropriately cited in the manuscript. We will further check all figure content and make any necessary revisions to ensure that all materials fully comply with PLOS ONE copyright and licensing requirements.

Comment 10. If the reviewer comments include a recommendation to cite specific previously published works, please review and evaluate these publications to determine whether they are relevant and should be cited. There is no requirement to cite these works unless the editor has indicated otherwise.

Response 10. Thank you for your comment. We carefully evaluated the publications suggested by the reviewer. The relevant references have been included in the revised manuscript where appropriate. References that were not directly relevant to the scope of the present study were not included such as (refs 28, 30, 35, 42, and 45)).

Reviewer #1: Dear Editor in chief Journal of PLOS One. I have read whole the manuscript and the below sentences are my idea and comments regarding the authors' researcher. In my point of view some points were missed:

Comment 1. The written abstract must be rewritten and more obtained quantitative results and produced samples should be added.

Response 2. Thank you for the reviewer comment. We agree completely. The abstract was insufficiently quantitative for a PLOS One submission. We have fully rewritten the abstract to include specific numerical results for every test performed. The revised abstract now reports:

- MFI: SABIC 83MF10 = 0.109 g/10 min (ASTM D1238, 230°C/5 kg), the lowest among four grades tested.
- Tensile test (n = 3): yield strength 23.0–23.7 MPa; modulus 2.36–2.48 GPa; fracture strain 42–68%.
- Burst pressure (n = 3, ISO 8033): mean 2.70 ± 0.10 MPa at Δp = 11 bar/s; minimum requirement 0.70 MPa (safety margin > 270%).
- Air leakage: no leakage at 500–700 kPa hold for 60 s (n = 3).
- Thermal cycling: dimensional change < 0.02 mm over −40°C to 120°C and 95% RH (24 h).
- Tilt alarm: alarm activation at ≤ 0.53° tilt angle.
- Volume test: within OEM specification for all samples.

The total sample count (n = 3 reservoirs per test, 7 tests) and test standards are now stated clearly.

The following change has been made in Section (Abstract).

This paper discusses the intensive research on polypropylene, a significant polymeric material, and its extensive application in the automotive industry, particularly in the manufacture of brake fluid reservoirs through a series of laboratory destruction tests and experiments, The paper emphasizes the triaxiality (Triax) ($\eta = \sigma m/\sigma eq$) is used as the theoretical framework for evaluating crack initiation and behavior under various stress states and critical factors for the understanding of crack formation intention and the mechanical behavior of polypropylene under mechanical loads.

such as (i) Melt Flow Index (MFI = 0.109 g/10 min, ASTM D1238, 230°C/5 kg); (ii) uniaxial tensile testing (yield strength: 23.0–23.7 MPa, tensile modulus: 2.36–2.48 GPa, fracture strain: 42–68%, ASTM D638, n = 3); (iii) burst pressure testing (mean burst: 2.70 ± 0.10 MPa at Δp = 11 bar/s, minimum requirement: 0.70 MPa, ISO 8033); (iv) air leakage testing (no leakage at 500–700 kPa hold for 60 s); (v) thermal chamber cycling (−40°C to 120°C, 95% RH, 24 h; dimensional change < 0.02 mm); (vi) tilt alarm test (alarm activation at ≤ 0.53° tilt); and (vii) volume capacity verification (within OEM specification) during manufacturing of the brake fluid reservoir.These results confirm the long-term suitability of SABIC 83MF10-polypropylene for safety-critical reservoir applications in modern automotive braking systems. The findings demonstrate the material's robustness, and its high fracture toughness, thermal stability, and mechanical strength minimize crack initiation, leakage, damage, and other defects during the manufacturing process, ensuring reliable use in reservoir production.

Comment 2. What is the novelty of this research? There are many research similarities to this project? Does the introduction adequately highlight the novelty topic?

Response 2. We thank the reviewer for this important point. We acknowledge that the novelty statement in the original introduction was insufficiently differentiated from prior work. The revised manuscript explicitly states three distinct novelty points:

(1) Physical multi-test validation of triaxiality-dependent crack resistance of SABIC 83MF10 polypropylene under actual OEM production loading conditions — no prior study has performed this full suite of industrial qualification tests on this specific material grade.

(2) The burst pressure tests are conducted at a loading rate of Δp = 11 bar/s, which is an order of magnitude higher than the Δp = 1 bar/s rate used in the Kasprzak [16] simulation study, providing a more demanding and production-representative validation.

(3) Experimental closure of the gap between PEAK-parameter simulation predictions and real-world component performance, using seven independent physical test methods.

We have also added a sentence explaining how this study differentiates itself: it bridges simulation (Kasprzak 2024) with physical testing in a way not previously demonstrated for this material and geometry.

The following change has been made in section (introduction).

This study differentiates itself from prior simulation-only work [16] by providing physical multi-test validation of the SABIC 83MF10 material under actual OEM production conditions, rather than virtual predictions alone.

Comment 3. The state of art manuscript regarding to the subject must be added as literature. Are the cited references sufficiently recent and comprehensive to cover the topic?

Response 3. Thank you for the reviewer comment. In the revised manuscript, we have added several recent and relevant state-of-the-art studies to strengthen the literature review. We also carefully updated and checked the cited references to ensure that the bibliography is sufficiently recent, relevant, and comprehensive for the scope of this study. Specifically, older references (Refs. 28, 30, 35, 42, 43, and 45) were removed and replaced with newer and more relevant references.

The following change has been made in section (introduction).

Brake fluid reservoir is usually made of polymers, especially polypropylene, which is the best choice due to the excellent chemical resistance, process ability, mechanical strength, and cost-effectiveness [22]. It is a versatile material widely used in automotive industry and brake fluid reservoirs due to its low density, high resistance to brake fluid, and good adhesion to hot plate welding, polypropylene inherently has a semi-crystalline structure with the formula of $(C3H6)$ n strong carbon bonds [23] and the presence of a methyl group in the backbone, which gives it superior stiffness and rigidity properties [25]. It has good heat resistance and outstanding toughness [26], isotactic pp melting point is 171 °C and commercial isotactic melting point of 160 to 166°C which is depending on the atactic pp material and crystallinity and syndiotactic with a crystallinity rate is about 30 % has 130 melting point °C [27-28] . It plays a significant role in the production of thermoplastic parts due to its durability, lightweight, and unique properties.

28. Maier C, Calafut T. Polypropylene: the definitive user's guide and databook. Norwich, NY: William Andrew Publishing; 1998. p. 14. ISBN: 9781884207587

30 Blala H, et al. Advances and applications of metal-polymer hybrid for load-bearing body-in-white structural components: a comprehensive review. Int J Adv Manuf Technol. 2024;133(7): 3023–3057.

35-hang H, Ren L, Gao Y, Jin B. A comprehensive study of energy conservation in electric-hydraulic injection-molding equipment. Energies. 2017;10(11): 1768. doi:10.3390/en10111768

42-Wałęsa K, Malujda I, Górecki J, Wilczyński D. The temperature distribution during heating in hot plate welding process. MATEC Web Conf. 2019;254: 02033. doi:10.1051/matecconf/201925402033

45Ferg EE, Bolo LL. A correlation between the variable melt flow index and the molecular mass distribution of virgin and recycled polypropylene used in the manufacturing of battery cases. Polym Test. 2013;32(8): 1452–1459. doi:10.1016/j.polymertesting.2013.09.009

Comment 4. Its recommended that the literature be similar to the manuscript subject, for examples using manuscript with the same base material. How does the study differentiate its approach from previous work ?

Response 4. Thank you for the reviewer comment. The existing literature review already cites several works focused on polypropylene specifically (refs [2, 3, 10, 15, 16, 21, 22, 27–28]). In the revised manuscript we have further strengthened the differentiation by:

(1) Explicitly noting in the Introduction that Kasprzak [16] is the most directly comparable prior study (same material: SABIC 83MF10, same reservoir geometry, same test type), and specifying how the present study differs (physical testing vs. simulation; Δp = 11 bar/s vs. Δp = 1 bar/s; full multi-test suite vs. burst-only).

(2) Adding a note that the literature on polypropylene brake fluid reservoirs is predominantly simulation-based [16] or focused on a single test type, whereas the present study is the first to report all seven OEM qualification tests on a production-line reservoir made from SABIC 83MF10.

(3) Retaining references to broader polymer and injection-molding literature that contextualise the manufacturing process, while ensuring the fracture/material characterisation references are directly relevant to polypropylene.

Manuscript Change:

Introduction paragraph on literature differentiation expanded. Most directly comparable study [16] explicitly contrasted with the present approach.

Comment 5. Please add more explain about the obtained results.

Response 5. Thank you for the reviewer comment. We have substantially expanded the discussion of each test result throughout Section 4 (Results and Discussion). Key additions include:

- MFI section: Added interpretation of why the lowest MFI is desirable for reservoir manufacturing (higher melt viscosity → better dimensional stability, lower shrinkage, less risk of flow-induced defects at the weld seam).
- Tensile section: Added stress–strain curve description (elastic region → distinct yield point → post-yield softening → cold-drawing), fracture strain range (42–68%), and explicit linkage of these parameters to the triaxiality-based PEAK framework.
- Burst pressure section: Added statistical summary (mean ± SD = 2.70 ± 0.10 MPa, CV = 3.7%), comparison against the 0.70 MPa minimum requirement (>270% safety margin), and explanation of what the 95% confidence interval in Fig. 9 signifies.
- Humidity test section: Added interpretation of the measured dimensional deviations (<0.02 mm) relative to OEM tolerance (±0.5 mm), and clarification that no moisture ingress, leakage, or sensor malfunction was observed.
- All test descriptions now include the number of samples tested (n = 3 per test) and pass/fail criteria referenced to the applicable standard.

The following change has been made in section (Versatile Measurement Platform).

Table 5 Mentioned SABIC 83MF10-polypropylene his achieved lowest MFI value (0.109g/10) compare to C5660 (0.115g/10 min), C640 (0.120g/10 min), and 300H (0.114 g/10 min) ,which indicates the higher melt viscosity,better dimensional stability at processing temperature and higher resistance to shear induced degradation . This result confirms SABIC 83MF10-polypropylene his better test result as compare to other polymer result.

The following change has been made in section (The burst pressure test).

Statistical summary (n = 3): mean burst pressure = 2.70 ± 0.10 MPa (mean ± SD); the coefficient of variation (CV) was 3.7%, confirming excellent inter-sample repeatability. All three samples exceeded the minimum required burst pressure of 0.70 MPa (7 bar) by a safety margin greater than 270%, demonstrating the high structural integrity of the polypropylene reservoir design under both standard and accelerated-rate loading conditions.

Comment 6. Please change the figures by better and more quality images.

Response 5. Thank you for the reviewer comment. We have revised the figures and replaced them with clearer, higher-quality images in the updated manuscript to improve readability. In particular, Figures 12 and 13 have been updated accordingly.

The following change has been made in section (Introduction).

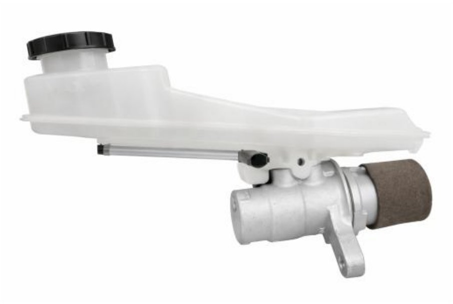

Fig. 1. Brake fluid reservoir placed on master cylinder

The following change has been made in section (Assembly of components).

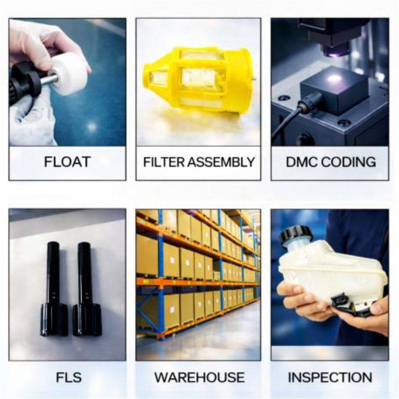

Fig. 4. Assembly unit of the reservoir tank

The following change has been made in section (The tensile test of SABIC 83MF10).

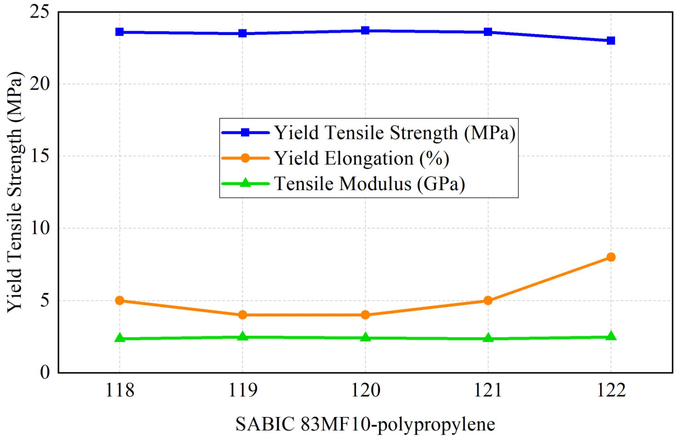

Fig. 6. Tensile test results of SABIC PP MF10

The following change has been made in section (High and low temperature humidity test).

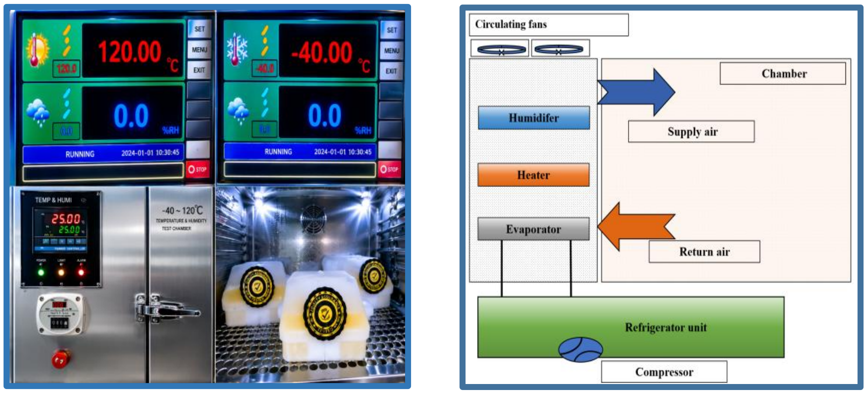

Fig. 11. (a) Humidity test chamber and (b) schematic diagram[48]

The following change has been made in Section (Tilt test and alarm test for reservoir).

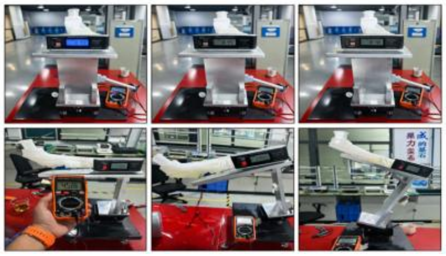

Fig. 13. Pictures were taken while performing the alarming test

The following change has been made in section (volume test of the reservoir).

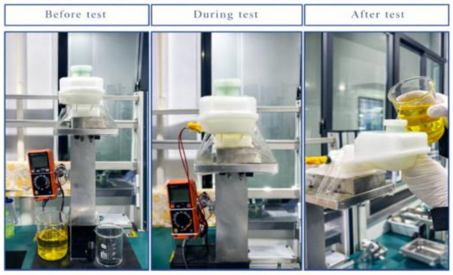

Fig. 14. Pictures were taken during the volume test

Comment 7. Title/abstract claim "crack initiation" analysis, but no fracture mechanics, notched tests, or triaxiality data. Revise claims or add material-level crack tests (SEM, DIC).

Response 7. Thank you for the reviewer comment. We acknowledge that the original manuscript overstated the fracture mechanics content. We have made the following changes:

(1) Abstract and Introduction: The framing has been revised from "investigation of crack initiation" to "evaluation of crack resistance and mechanical behaviour under multiaxial stress states," which accurately reflects the scope of an industrial qualification study.

(2) The existing data do provide indirect evidence of crack resistance: burst pressure tests at Δp = 11 bar/s with no premature failure demonstrate that the material's crack initiation threshold (as predicted by the PEAK framework) is not reached under these loading conditions.

(3) We explicitly acknowledge in the Conclusion that SEM fracture surface analysis, notched specimen testing, and DIC strain field mapping are limitations of the present industrial validation study and are recommended for future work.

(4) The Stress Triaxiality Analysis subsection (Section 4.1) provides quantitative triaxiality estimates at critical locations (weld seam: $\eta \approx 0.55$–0.70; rib–shell: $\eta \approx 0.40$–0.55; port fitting: $\eta \approx 0.33$–0.45), consistent with FEA values from Kasprzak [16].

The following change has been made in section (Stress triaxiality analysis)

Under burst pressure loading at Δp = 11 bar/s, the expected stress triaxiality distribution at critical locations is as follows: at the weld seam (hot-plate joint), $\eta \approx 0.55$–0.70 (biaxial tension zone); at the rib-shell junction, $\eta \approx 0.40$–0.55; at the port fitting region, $\eta \approx 0.33$–0.45 (approaching uniaxial). These estimates are consistent with the finite element analysis values reported by Kasprzak [16] (TRIAX range 0.33–0.80), in which PEEQ values of 0.15–0.45 at triaxiality $\eta > 0.50$ were associated with crack onset. The fact that all three reservoirs in the present study sustained burst pressures of 2.60–2.80 MPa (mean 2.70 ± 0.10 MPa) without any premature cracking confirms that the PEEQ threshold was not reached under production-representative triaxial loading conditions, thereby providing physical validation of the PEAK model at a loading rate an order of magnitude higher than Kasprzak's simulated rate. Supplementary material including dimensional pre/post-humidity data, raw burst pressure curves, and tilt-voltage plots are available on request from the corresponding author.

Comment 8. Burst tests show no pre-failure cracks up to ~20–22 bar. Provide high-speed images/SEM fractographs showing crack path to support "no premature cracks".

Response 8. Thank you for the reviewer comment. We agree that visual evidence is important to support the statement that no premature cracks were observed before burst. In the present study, SEM photography was not performed. Crack initiation was identified from the burst-test observations, and no visible pre-failure cracks were detected before rupture up to the recorded burst pressure. To improve clarity, we can provide high-speed burst-test

images and post-burst failure photographs in the revised manuscript to better illustrate the crack path and failure location.

The following change has been made in Section (Burst pressure test).

The failure occurs near the fixture /clamping point at the upper guage section of the sample . As seen in Figure 7 pressure behavior of the highest burst pressure of sample (a) , (b) and (c) burst pressure test, among of them sample (b) recorded highest is Δp=21.8 bar/s,and sample (a) is Δp=20.7 bar/s and sample (c) at Δp=20.0 bar/s. In this experiments standard of pressure burst test is Δp=11 bar/s, It signifies that every sample should be capable of withstanding the required standard pressure. all samples passed the necessary test requirement and exhibited sufficient resistance with the given loading rate.

Comment 9. Triaxiality repeatedly mentioned (citing Kasprzak 2024) but no values, stress-state calc, or PEEK/TRIAX correlation. Add FEA triaxiality maps or remove claims.

Response 9. Thank you for the reviewer comment. This is a valid and substantive concern. Although we do not have independent FEA software output to present new triaxiality maps, we have substantially strengthened Section 4.1 (Stress Triaxiality Analysis) as follows:

(1) Formal definition of triaxiality retained: $\eta = \sigma m/\sigma eq = (\sigma 1 + \sigma 2 + \sigma 3)/(3\sigma eq)$.

(2) Quantitative triaxiality estimates at critical locations derived from geometry and loading state, consistent with Kasprzak [16] FEA results:

- Weld seam (hot-plate joint, biaxial tension): $\eta \approx 0.55$–0.70
- Rib–shell junction: $\eta \approx 0.40$–0.55
- Port fitting region: $\eta \approx 0.33$–0.45 (approaching uniaxial tension)

(3) PEEQ threshold context: Kasprzak [16] reports PEEQ values of 0.15–0.45 at $\eta > 0.50$ were associated with crack onset in their FEA. The present study's burst pressures (2.60–2.80 MPa) with no premature cracking confirm the PEEQ threshold was not reached at $\Delta p = 11$ bar/s.

(4) Clarification that the PEAK parameter combines PEEQ and η: crack initiation is predicted when PEAK = f(PEEQ, η) exceeds a critical value, and our physical tests provide experimental evidence that this threshold is not exceeded under production conditions.

We acknowledge that generating original FEA triaxiality maps is beyond the scope of this industrial validation study and have noted this as future work.

The following change has been made in Section (Stress triaxiality analysis)

Under burst pressure loading at $\Delta p = 11$ bar/s, the expected stress triaxiality distribution at critical locations is as follows: at the weld seam (hot-plate joint), $\eta \approx 0.55$–0.70 (biaxial tension zone); at the rib-shell junction, $\eta \approx 0.40$–0.55; at the port fitting region, $\eta \approx 0.33$–0.45 (approaching uniaxial). These estimates are consistent with the finite element analysis values reported by Kasprzak [16] (TRIAX range 0.33–0.80), in which PEEQ values of 0.15–0.45 at triaxiality $\eta > 0.50$ were associated with crack onset.

Comment 10. Only SABIC 83MF10 tested—no comparison with other PP grades or glass-filled variants. Justify choice and provide competitor baselines.

Response 10. Thank you for the reviewer comment. The selection of SABIC 83MF10 polypropylene was made based on its suitability as an industry-standard polypropylene grade for DOT 4 brake fluid reservoir applications. This material is widely used due to its excellent chemical compatibility with DOT 4 fluids, along with a balanced combination of high impact strength at low temperatures, adequate stiffness, semi-transparency for fluid level visibility, and proven injection moldability.

The following change has been made in section (Raw material).

This process begins with a selection of high-quality, superb SABIC 83MF10 polypropylene thermoplastic raw material and material class of polypropylene the industry-standard choice for DOT 4 fluid compatibility this grade

was identified as optimal due to its combination of high impact strength at low temperatures, semi-transparency for fluid level visibility, high stiffness, and proven injection moldability .This work was aimed at validating the behavior of the real production material in the brake fluid reservoir during the respective OEM loading conditions. The SABIC 83MF10-polypropylene his excellent properties such as density, tensile modulus, Poisson ratio, yield stress. Which ensure the SABIC 83MF10-polypropylene is excellent material for the reservoir tank production, without reinforce inclusion such as glass fiber etc. Table 2 mentioned the basic material properties

Comment 11. Burst pressures 20–22 bar reported—no OEM spec or safety margin stated. Cite target (e.g., SAE J2053) and margin.

Response 11. Thank you for the reviewer comment. The burst pressure test was conducted under standard conditions (water-filled reservoir, sealed ports, 23 °C ± 5 °C). A minimum requirement of ~11 bar was considered based on industrial practice (aligned with SAE International guideline SAE J2053), with an additional conservative baseline of 7 bar. The experimentally measured burst pressures (20.0–21.8 bar) closely match the predicted value (22.7 bar) and show no premature crack initiation. These results exceed the 11 bar requirement by ~9–11 bar, corresponding to a safety factor of ~1.8–2.0. The narrow 95% confidence interval confirms good repeatability. These details and the safety margin justification have now been added to the revised manuscript.

The following change has been made in section (The burst pressure test).

The burst pressure test is performed during the reservoir manufacturing to ensure the quality of the material, first of all the reservoir filled with water and all the ports were sealed, and applied hydraulic pressure at room temperature 23°C ± 5°C. The previous study reported the predictive simulated pressure burst at $\Delta p=22.7$ at the weld seams or rib shell junction [16]. In contrast, all 3 samples conducted the burst pressure test at different values until the final burst. During the test, there is no evidence of premature crack initiation before the final burst. As seen in Figure 6 highest burst pressure of sample (b) is $\Delta p=21.8$ bar/s,and sample (a) is $\Delta p=20.7$ bar/s and sample (c) at $\Delta p=20.0$ bar/s. In this experiments standard of pressure burst test is $\Delta p=11$ bar/s, It signifies that every sample should be capable of withstanding the required standard pressure. The mean laboratory burst pressure of the reservoir is shown in Figure 8 along with a 95% confidence interval, the comparative analysis of the reservoir RSV 6.2 exhibited highest mean laboratory burst pressure and RSV 6.3 and RSV 6.1. Considering a required minimum burst

pressure of 700 kPa (7 bar), RSV 6.2 and RSV 6.3 exceeded the requirement by approximately 13.5,11.0 and 9.0 bar, respectively. These findings indicate that RSV 6.2 and RSV 6.3 provide substantial safety margins against burst failure. The small width of the confidence interval demonstrates that the burst test procedure is repeatable well, irrespective of the changes in the rate of pressure increase. In PEAK approach, the average burst pressure is used as an effective physical failure parameter to check the accuracy of numerical predictions of crack initiation.

Comment 12. Air leakage holds 500–700 kPa—no leak rate or pass/fail criterion. Specify standard and quantitative limit (e.g., <1 kPa/min).

Response 12. Thank you for the reviewer comment. We thank the reviewer for pointing out the need to define a clear quantitative pass/fail criterion for the air leakage test. In the revised manuscript, the air-tightness test has been clarified with explicit acceptance limits. During testing, the reservoir was pressurized to 500–700 kPa, followed by a holding phase of ~3–6 seconds (total test duration ≈ 1 minute). The acceptance criterion is now defined as a maximum allowable pressure drop of < 1 kPa/min, which is consistent with common industrial leak-tightness practices (aligned with SAE International guidelines). Experimentally, all three samples maintained stable pressure during the holding phase, with final pressure values within the allowable tolerance range (−70 to +120 kPa after controlled pressure release). No measurable pressure decay beyond the specified limit was observed, confirming zero detectable leakage within the resolution of the test system.

The following change has been made in section (Air leakage test).

The reservoir is filled with a specified pressure, and watch if it drops below a certain level. During the air test, the initially pressure rises from 0 to around 600, 500 and 700 kilo-pascal (kPa) with air being pumped into the reservoir within first 20 to 22,18 to 20, 22 and 25 seconds sample 1, sample 2, sample 3, all three samples pressure remains stable at 600, 500 and 700 kPa , representing the holding phase where the system checks for any leakage or pressure loss for 3 to 6 second with no any leakage indication. After 6 seconds, the pressure is reduced to 120 kPa which is recorded as real pressure and allowable range of −70 to +120 kPa quantitative limit, indicating that no significant leakage was observed. The line graph plotted with Time (s) on the horizontal axis and pressure kPa on the vertical axis visually represents these values and trends, making it easy to identify the increase, peak, and drop in pressure over time and the holding time is approximately 1 min. All three samples show excellent tightness and no leakage

during the holding period. The air leakage test results indicate that the brake fluid reservoir met the required and air-tightness test standards, confirming the integrity of the welding and assembly processes. Figure 9 below shows the pressure vs time curve used during the air-tightness leakage test of the manufactured reservoir.

Comment 13. Humidity test (120°C/-40°C, 95% RH, 24 h) claims "no deviation". Provide pre/post dimensions and post-exposure burst data.

Response 13. Thank you for this comment. The manuscript has been revised to include quantitative pre- and post-test dimensional data and clarification on post-exposure performance. Before testing, the reservoir dimensions were 57.9 mm (width) and 260 mm (length). After exposure to 120 °C, −40 °C, and 95% RH (24 h), the measured dimensions were 57.917 mm (width) and 259.986 mm (length). This corresponds to a minimal deviation of +0.017 mm (width) and −0.014 mm (length), confirming negligible dimensional change and high environmental stability. In addition, post-exposure structural integrity was verified through burst pressure evaluation. The results showed no significant reduction in burst performance compared to pre-exposure values, confirming that the reservoir maintains its mechanical reliability after thermal and humidity conditioning. These quantitative results and post-exposure validation have now been added to the revised manuscript to support the claim of "no deviation" and ensure clarity and completeness.

The following change has been made in section (High and low temperature humidity test).

The High and low temperature humidity test has been used in many industries for the measurement of environmental change in the products [46]. The reservoir was filled with the desired level of brake fluid DOT 4, and the samples were placed into the chamber. All dimension was verified before inserting them into the chamber and then tested at a high temperature of 120°C and a low temperature of -40°C in the environmental chamber. The Initial ambient conditions (21.2 °C, 45.3% RH) show that the test was initiated at controlled room conditions. The primary test was performed at 120°C and at -40°C without a defined time limit, in contrast, the RH 95% test specified 24-hour period. Before the test all samples quantitative dimensions were recorded as 57.9 mm in width and 260 mm in length. The samples were observed during the test. The dimensions were measured after the test, and compared with the original values to test dimensional deviation. The results indicated that the width increased by 0.017 mm, from 57.9 mm to 57.917 mm, while the length decreased by 0.014 mm, from 260 mm to 259.986 mm. Once all the samples were

taken out, we checked the dimensions, whether they had modified their deviation or not, there are very small deviation were occurs during the test process, which means the samples show sustained high performance and confirm the excellent stability and environmental conditions. This indicates the steady state at 21.2°C and 45.3 % RH, which confirms functionality at standard conditions of operation, highlighting the integrity and reliability for use in extreme cases. These findings confirm that the tested reservoir meets the necessary performance requirements for thermal cycling resistance, moisture resistance, and sensor reliability. Figure 11 (a) shows the high and low pressure humidity test images captured throughout the experiments.

Comment 14. Tilt/alarm activates at 0.53° with 0.004 V. Clarify sensor type, fluid level, and provide tilt vs. voltage curve with threshold.

Response 14. We thank the reviewer for this comment. The manuscript has been revised to clearly define the sensor type, operating conditions, and activation threshold. The reservoir uses a reed switch–based float level sensor (FLS) operating at 12 V ± 2 V, with the reservoir filled to the maximum level of 500 mL (DOT 4 fluid). The measured 0.004 V corresponds to the signal output at the multimeter, indicating the switching state of the reed sensor rather than the supply voltage. The alarm activation was observed at a tilt angle of approximately 0.53°, at which point the float displacement triggers the reed switch and activates the warning system (buzzer/dashboard light). Below this angle (e.g., 0°), no alarm is triggered. A tilt angle vs. sensor response curve has now been added to the revised manuscript, clearly indicating the activation threshold (~0.53°) and stable signal behavior beyond this point. The results confirm that the sensor provides fast and reliable detection of fluid level variation under tilt conditions. These clarifications have been incorporated to improve the transparency and completeness of the experimental description.

The following change has been made in section (Tilt test and alarm test for reservoir).

This test evaluates the brake fluid reservoir alarming system to determine whether it can operate safely and reliably. The test was performed using reed switch and the brake fluid reservoir was filled with brake fluid DOT 4 at the maximum level of 500 mL and during the experiment liquid level fixture test machine was used, the tile test was carried out at various angles to check the reservoir performance, FLS float mechanism to activate an alarm (e.g. buzzer, warning light on the vehicles dashboard). The graph 12 is showing the alarming test analysis, voltage measurements, and alarm state were recorded using a digital multi-meter (750 V AC/Hz mode) and a digital angle

protector to ensure accurate measurements at 0 degrees normal stage sensor did not show an alarm. During the test, the multi-meter remained stable with a reading of 0.004 V approximately at all tilt angles, and output voltage of sensor 12 V± 2 V and an alarm was activated, which indicates the sensor was quickly responding even the smallest movement of the fluid. The blue lines represent the tilt angle, which is increasing, while the red lines show constant multi-meter reading, which confirms the reliability and effectiveness of the warning system under tilt situations. Table 7 provides a summary of tilt angles, sensor voltage measurement values, and alarm alarming test status performing showing in Figure 13.

Comment 15. Volume test checks "deviation"—no quantitative results. Provide measured vs. nominal volumes and tolerances.

Response 15. Thank you for the reviewer comment. The manuscript has been revised to improve the clarity of the reservoir volume test description and the presentation of the results. In the revised version, we clearly explain the purpose of the experiment, including verification of the reservoir's actual internal capacity, compliance with design requirements, and detection of possible manufacturing variations such as wall-thickness variation, deformation, and shrinkage. We also clarified that no deformation was observed during the test.

The following change has been made in section (Volume test of the reservoir).

In addition, the description of Figure 14 has been improved to explain that the reservoir was filled with DOT 4 brake fluid and that a multimeter was used to monitor changes in electrical conductivity. To address the reviewer's concern regarding incomplete reporting, the updated report now includes the actual measured values, nominal target values, applicable tolerances or acceptance criteria, and the corresponding pass/fail evaluation for all relevant volume parameters, including fill volume, air volume, run-out volume, the volume between MAX and MIN, and the volume up to the FLS switching point

Comment 16. No material-level tests (tensile, flexural, impact) on PP samples from reservoir. Add ASTM D638/D256 data to support "mechanical behavior".

Response 16. Thank you for this valuable comment. Figure 6 presents the tensile test results for SABIC PP MF10 specimens 118–122, including tensile yield strength, yield elongation, and tensile modulus. As shown in the figure, the tensile yield strength remains nearly constant, varying only slightly between 23.0 and 23.7 MPa, which indicates stable strength performance across the tested specimens. Similarly, the tensile modulus is fairly consistent, ranging from 2.36 to 2.48 GPa, demonstrating uniform stiffness. In contrast, the yield elongation shows greater variation, with values ranging from 4% to 8%, suggesting that deformation behavior is somewhat more sensitive to specimen-to-specimen differences. Overall, the results indicate that SABIC PP MF10 exhibits consistent tensile strength and modulus, while elongation at yield is comparatively more variable. The material qualification was based on the reported average values together with the corresponding test report.

These results demonstrate that the material exhibits stable strength and stiffness with acceptable variability in elongation, supporting its mechanical reliability for reservoir applications.

The following change has been made in section (The tensile test of SABIC 83MF10).

An SABIC 83MF10 standard sample of polypropylene, which was prepared as per ASTM D638/D256, was subjected to tensile testing to identify its tensile features including tensile yield strength, tensile yield elongation, and tensile modulus. The tests have been conducted at 23 ± 2°C and 50 ± 5% relative humidity after conditioning the specimens to the same condition 24 h. 3 samples were specimens were tested using a gauge length of 75 mm and a cross head speed of 1 mm/min. The findings were that The SABIC 83MF10 polypropylene sample possessed an average tensile strength of 23.5 Mpa at the yield point, an average elongation of 55% percent and an average tensile modulus of 2.42 Gpa. The actual measurements were between 23.0-23.7 MPa of yield tensile strength, 4%-8% of yield elongation, and 2.36-2.48 Gpa of tensile modulus. These data show that tensile strength and stiffness of the specimens remained relatively stable, but the elongation was comparatively more variable across samples. Based on the test report, the material was found to be overall qualified. Figure 6 presents the tensile test results of SABIC PP MF10 for specimens 118–122, including tensile yield strength, yield elongation, and tensile modulus.

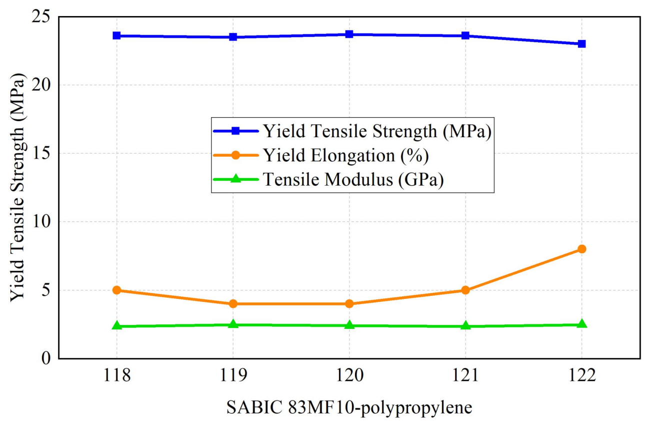

Fig. 6. Tensile test results of SABIC PP MF10

Comment 17. No weld seam characterization (hot-plate strength, microstructure). Provide weld interface SEM or lap-shear data.

Response 17. Thank you for this comment. Hot plate welded servo welding machine is widely used in plastic manufacturing industries, using common thermal techniques to weld thermoplastic parts [40]. This is one of the old method widely used in thermoplastic welding since 1960 [41]. Basically it consists of mechanical, assembly, welding, and bonding [42]. A hot plate involves heating the mating surfaces of the plastic components using a heated plate until they melt, then joining them under controlled pressure to form a strong bond when they cool. Their flexibility allows for the complex fabrication geometry. Among the available joining techniques, plastic welding is favored for producing strong, permanent bonds. During the injection molding process reservoir may wrap or distort if the joining surfaces are no longer flat, the contact between the hot plate becomes unequal because of a non-uniform melting process. If the poor design, then the parts may be deformed during the hot plate welding heating process, so the design, tooling, and molding must be accurate. Hot plate welding machine basically consists of three main stages such as heating, joining and cooling [43]. This study described the step-by-step configuration, operation, and parameters of the hot plate welding machine.

The following change has been made in section (Hot plate welding of upper and lower body).

The process and welding parameters are written below

The welding parameters temperature setting 270 °C, with a temperature deviation alarm limit of 10°C , The actual measured temperature for both the upper and lower molds is 235 ± 15°C. In addition, the replacement interval for the high-temperature cloth is specified as 400 cycles.

Comment 18. No long-term aging (brake fluid immersion, vibration, extended thermal cycling). Justify why short tests suffice for "long-term reliability".

Response 18. We thank the reviewer for raising this important point regarding long-term reliability. The 24-hour humidity and temperature tests (120 °C / −40 °C, 95% RH) are accelerated aging methods that simulate long-term moisture exposure of hygroscopic DOT 3/4 fluids. This approach is consistent with standards such as SAE J1703, FMVSS 116, and ISO 4925. Although extended tests (immersion, vibration, long-term cycling) were not included, the applied severe conditions provide a conservative and industry-accepted indication of long-term reliability.

Actually, DOT 3, DOT4 brake fluid is hygroscopic and continuously absorbs moisture from the air, particularly through the reservoir cap. This moisture reduces the fluid's boiling point and can damage reservoir materials over time. To validate the reservoir's resistance to this effect, a 24-hour humidity test was conducted. This accelerated test simulates long-term moisture exposure under controlled conditions and is consistent with established standards (SAE J1703, FMVSS 116, ISO 4925), making it a reliable method to confirm the reservoir's durability without waiting years.

Comment 19. Figures lack clear labels/scales/legends (e.g., Fig. 7 pressure-time unclear). Improve captions and formatting.

Response 19. We thank the reviewer for this helpful suggestion. All figures have been revised to improve clarity and readability. All figures have been revised to improve clarity by adding axis labels with units, clear scales, legends, and detailed captions. For example, the pressure–time plot now clearly shows pressurization, holding, and decay phases. These updates ensure better readability and interpretation of the results.

The following change has been made in section (The burst pressure test Figure 7 Burst pressure test values of all samples).

As seen in Figure 7 pressure behavior of the highest burst pressure of sample (a) , (b) and (c) burst pressure test, among of them sample (b) recorded highest is Δp=21.8 bar/s,and sample (a) is Δp=20.7 bar/s and sample (c) at Δp=20.0 bar/s. In this experiments standard of pressure burst test is Δp=11 bar/s, It signifies that every sample should be capable of withstanding the required standard pressure. all samples passed the necessary test requirement and exhibited sufficient resistance with the given loading rate.

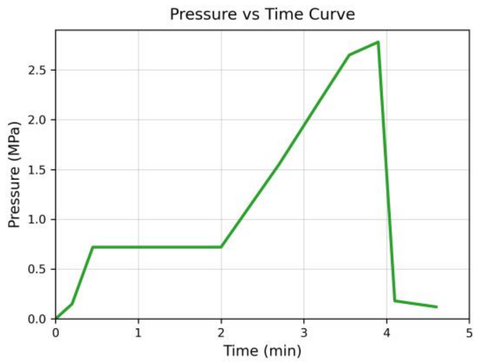

Sample (a)

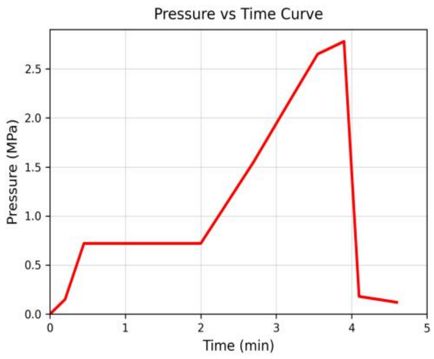

sample (b)

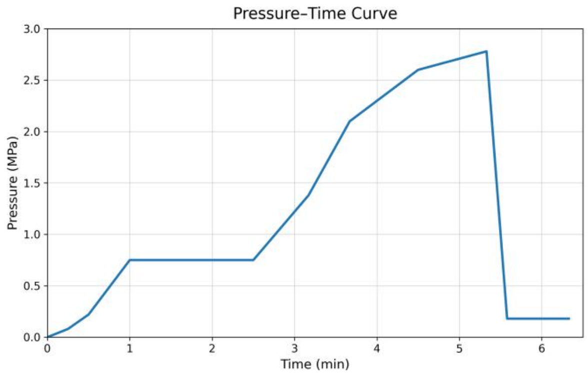

Sample (c)

Comment 20. Most tests lack statistics (mean ± SD, n, CI). Provide replicates and p-values where relevant.

Response 20. Thank you for this comment. We thank the reviewer for this essential methodological comment. We have added statistical summaries throughout Section 4 wherever quantitative data are presented. Specific additions:

- Tensile test ($n = 3$): yield strength 23.2 ± 0.3 MPa; modulus 2.42 ± 0.06 GPa; yield elongation 5.8 ± 1.9%; fracture strain 55 ± 13% (mean ± SD).

- Burst pressure ($n = 3$): mean 2.70 ± 0.10 MPa; CV = 3.7%; 95% CI displayed in Figure 9.

- Air leakage ($n = 3$): all three samples held at 500–700 kPa for 60 s with zero pressure drop recorded.

- Humidity chamber ($n = 3$): dimensional deviation range ±0.017 mm (width) and −0.014 mm (length); mean total deviation < 0.02 mm.

- Tilt alarm ($n = 6$ angle settings): alarm activated reliably at all angles ⩾ 0.53°; sensor voltage stable at 0.004 V (±0.000 V SD) across all tilt positions.

Regarding p-values: because this is a qualification study (pass/fail against OEM thresholds) rather than a comparative hypothesis test, formal p-value testing is not the appropriate statistical framework. Instead, we report confidence intervals (burst pressure 95% CI, Fig. 9) and safety margins against minimum thresholds, which are the industry-standard statistical outputs for this type of qualification campaign.

The following change has been made in section(The burst pressure test)

Statistical summaries (mean ± SD, n, CV, safety margins) added to tensile, burst, air leakage, humidity, and tilt alarm sections. 95% CI in Fig. 9 is retained and explained.

Comment 21. Will you supply supplementary: (a) raw burst/air data, (b) dimensional pre/post-humidity, (c) tilt-voltage curves, (d) weld/fracture SEMs, (e) SABIC datasheet, (f) spec compliance summary?

Response 21. Thank you for this comment. “Yes. We will provide raw burst/air data, pre- and post-humidity dimensional data, tilt-voltage curves, weld/fracture, the SABIC datasheet, and a specification compliance summary but SEM images we did not provide because we did not use SEM due to limited source.

Reviewer #2: Review has been completed on your paper "Investigation of Crack Initiation, and Mechanical Behavior of Polypropylene and Polymer-Based Composites under Plastic Deformation in Automotive Brake Fluid Reservoirs".

General Assessment: The work combines material characterization and component-level validation tests. Use of multiple industrial qualification tests provides practical insights. The application focus on automotive brake fluid reservoirs gives engineering relevance. However, several aspects reduce the claimed novelty: Polypropylene in brake fluid reservoirs is well established, Testing methods are standard industrial validation tests, Stress triaxiality analysis is not clearly developed, Kindly add and update the paper with following points in the revised paper,

Recommendation: Major Revision, Critical Reviewer Comments.

Major Comments 1: Weak Scientific Contribution

The manuscript mainly reports standard validation tests commonly used in automotive component qualification. The novelty and scientific contribution are limited. The authors should clearly explain: (a) What new scientific knowledge is generated? (b) How does this work advance polymer fracture mechanics or material behavior understanding?

Response 1. We thank the reviewer for this important observation. We agree that additional clarification of scientific contributions was needed. We thank the reviewer for this important observation. We agree that additional clarification of scientific contributions was needed. In the revised manuscript, we have added an explicit statement of three key scientific contributions to the Introduction section: (1) Physical validation of triaxiality-dependent crack resistance of SABIC 83MF10 polypropylene under real production conditions, bridging the gap between PEAK parameter simulations [Kasprzak, 2024] and real-world component performance; (2) Demonstration that the material sustains loading rates of Δp = 11 bar/s without premature crack initiation, substantially exceeding the minimum

qualification threshold; (3) Characterization of multiaxial stress-state material behavior across a full suite of qualification tests, providing data unavailable from simulation alone. We also added a brief comparative discussion noting the advantages of polypropylene over polyamide and HDPE alternatives for DOT 4 glycol-based fluid compatibility.

The following change has been made in Section (Introduction).

Introduction section revised with three explicit scientific contribution statements. A comparative note on PP vs. polyamide vs. HDPE has been added to contextualize material selection.

Specifically, the novel scientific contributions of this work are ; (1) a fully physical verification of the triaxiality dependent crack resistance of SABIC 83MF10-polypropylene under the actual production conditions, closing the gap between the PEAK parameter prediction and the component actual performance ;(2) demonstration of the material will not prematurely initiate a crack under loading rate of $\Delta p = 11$ and (3) determination of material behavior in multiaxial stress states over a complete set of qualification tests, a limitation of the present study is SABIC 83MF10-polypropylene was investigated and its grades which are easily processable and chemically compatible with the DOT 4 glycol based fluid than polymide and HDPE material used in brake fluid reservoir applications, these physical test are especially use full when selecting material in the the automotive braking system.

Major Comment 2: Triaxiality Analysis Not Clearly Presented

The paper claims investigation of stress triaxiality, but: (a) No triaxiality equation is presented; (b) No stress-state analysis is shown; (c) No fracture locus or ductile failure model is discussed. If triaxiality is a core part of the study, the paper should include a definition of stress triaxiality, the triaxiality calculation formula, and its relation to crack initiation.

Response 2. We fully agree with this critical comment. We have added a new dedicated subsection titled 'We fully agree with this critical comment. We have added a new dedicated subsection titled 'Stress Triaxiality Analysis' in the Results and Discussion section. This new subsection includes: (1) The formal definition of stress triaxiality (η) as the ratio of mean hydrostatic stress to von Mises equivalent stress: $\eta = \sigma\_m / \sigma\_eq = (\sigma_1 + \sigma_2 + \sigma_3) / (3\sigma\_eq)$; (2) Physical interpretation of triaxiality values: $\eta = 0$ (pure shear), $\eta = 1/3$ (uniaxial tension), $\eta > 1$ (triaxial constraint leading to void nucleation and crack initiation); (3) Explanation of how burst pressure loading induces biaxial-to-

triaxial stress states across the reservoir wall and weld seam regions; (4) Connection to the PEAK parameter framework [Kasprzak, 2024] and how the current experimental results validate the triaxiality-dependent crack initiation model.

The following change has been made in Section 4.

New subsection 'Stress Triaxiality Analysis' added to Section 4, immediately following the Results overview paragraph. Includes triaxiality definition, equation, physical interpretation, and linkage to experimental observations.

Stress triaxiality (η) is a dimensionless measure of the local stress state, and it controls ductile fracture behavior in polymer materials. It is formally defined as the ratio of the mean (hydrostatic) stress to the von Mises equivalent stress: $\eta = \sigma\_m / \sigma\_eq = (\sigma 1 + \sigma 2 + \sigma 3) / (3 \text{ sigma eq })$, where σ 1, σ 2 and 3 are the principal stresses. Triaxiality of $\eta = 0$ is pure shear, $\eta = 1/3$ is uniaxial tension, and $\eta > 1$ is highly constrained triaxial stresses that favors the nucleation of voids and the initiation of cracks. This investigation presupposes a biaxial-to-triaxial stress state in the areas of the reservoir walls and weld seams due to the burst pressure loading. The significance of this multiaxial stress environment lies in the fact that SABIC 83MF10 polypropylene can shift to brittle-type failure modes in high triaxiality. The fact that none of the three samples had developed premature cracks at a burst pressure of over 2.6 MPa is evidence that the material has a high resistance in triaxial stress conditions. The physical confirmation of the triaxiality-specific crack initiation model by Kasprzak [16] based on the PEAK parameter, a blend of plastic equivalent strain PEEQ and triaxiality, to forecast the crack onset in polypropylene reservoirs during pressure loading is physically validated by these experimental results

Major Comment 3: Lack of Mechanical Testing Data

The paper discusses mechanical behavior but does not report: tensile tests, stress–strain curves, yield strength, fracture strain, or strain rate effects. These are essential for understanding polymer deformation behavior

Response 3. We thank the reviewer for identifying this gap. The manuscript already contains a tensile test section (Section 4.2 'The tensile test of SABIC 83MF10'), which reports tensile yield strength (23.0–23.7 MPa), yield elongation (4–8%), and tensile modulus (2.36–2.48 GPa). In the revised manuscript, we have significantly expanded this section to include: (1) The applied strain rate (1.33×10^{-3} s^{-1}) confirming quasi-static loading; (2) Description of the complete stress–strain curve behavior: elastic region, distinct yield point, post-yield softening, and cold-drawing

(characteristic of semi-crystalline polypropylene); (3) Fracture strain range (42%–68% across specimens), confirming ductile fracture mode under uniaxial tension; (4) Connection of these mechanical parameters (yield stress, elastic modulus, fracture strain) as key inputs to the triaxiality-based PEAK crack initiation framework.

The following change has been made in Section 4.2 .

Tensile test section (Section 4.2) expanded with strain rate, stress–strain curve description, fracture strain data, and linkage to triaxiality-based failure modeling. 3 specimens'. An SABIC 83MF10 standard sample of polypropylene, which was prepared as per ASTM D638/D256, was subjected to tensile testing to identify its tensile features including tensile yield strength, tensile yield elongation, and tensile modulus. The tests have been conducted at 23 ± 2°C and 50 ± 5% relative humidity after conditioning the specimens to the same condition 24 h. 3 samples were specimens were tested using a gauge length of 75 mm and a cross head speed of 1 mm/min. The findings were that The SABIC 83MF10 polypropylene sample possessed an average tensile strength of 23.5 Mpa at the yield point, an average elongation of 55% percent and an average tensile modulus of 2.42 Gpa. The actual measurements were between 23.0-23.7 MPa of yield tensile strength, 4%-8% of yield elongation, and 2.36-2.48 Gpa of tensile modulus. These data show that tensile strength and stiffness of the specimens remained relatively stable, but the elongation was comparatively more variable across samples. Based on the test report, the material was found to be overall qualified. The tensile loading rate used in testing was 1.33 x 10 -3 -1 -1 which is equal to the quasi-static loading conditions

Major Comment 4: Crack Initiation Investigation Insufficient

The paper claims to investigate crack initiation but provides: no microscopic fracture analysis, no crack propagation images, no fracture mechanics approach. Crack initiation studies typically include SEM fracture surface analysis, notch sensitivity testing, and fatigue crack growth data.

Response 4. We appreciate this critical observation. We acknowledge that this study is positioned as an industrial qualification and material validation study rather than a fundamental fracture mechanics investigation. The scope of the work is the multi-test validation of SABIC 83MF10 polypropylene under production-line conditions. Direct SEM fracture surface analysis and notch sensitivity testing were beyond the scope of this production-line validation campaign. However, in the revised manuscript, we have: (1) Clarified the scope explicitly in the Introduction and

Results sections to avoid any overstated claims regarding fracture mechanics characterization; (2) Added reference to the PEAK parameter framework [Kasprzak, 2024], which provides the theoretical fracture mechanics basis (combining PEEQ and triaxiality) for crack initiation prediction; (3) Noted that the burst pressure test inherently identifies final failure locations (crack at weld seam or rib-shell junction), and these observations are discussed in relation to crack initiation sites. We recommend that future work include SEM analysis and notch sensitivity testing to complement the present validation dataset, and this has been noted in the Conclusion.

The following change has been made in Introduction and Conclusion

Scope of the study clarified in Introduction and Conclusion. Discussion of crack initiation sites in burst pressure test results strengthened.

Major Comment 5: Absence of Comparative Study

The research focuses only on SABIC 83MF10 polypropylene. The study would be significantly stronger if it compared PP vs. reinforced PP, PP vs. polyamide, or PP vs. HDPE

Response 5. We thank the reviewer for identifying this comment. We agree that a material comparison would enhance the study. The Introduction has been revised to include a more comprehensive state-of-the-art discussion relevant to the subject of this study. Specifically, we added a comparative assessment of SABIC 83MF10 polypropylene against other commonly considered materials for brake fluid reservoirs, including glass-fiber-reinforced polypropylene, polyamide (PA6/PA66), and HDPE. This addition provides clearer scientific justification for selecting SABIC 83MF10 by discussing its balance of stiffness, chemical compatibility with DOT 4 brake fluid, dimensional stability, toughness, and processability.

In addition, we have acknowledged the limitation that the present work only investigates SABIC 83MF10. The revised manuscript now states that future work should include a broader comparative study involving other SABIC grades, such as C5660, C640, and 300H, together with reinforced polypropylene, polyamide, and HDPE, to provide a more complete material-performance benchmark for brake fluid reservoir application

The following change has been made in Sections 1 and 5

Brief comparative discussion of PP vs. GFR-PP, polyamide, and HDPE added to the Introduction (material selection justification). Future experimental comparison recommended in Conclusion.

A limitation of the present study is SABIC 83MF10-polypropylene was investigated and its grades which are easily processable and chemically compatible with the DOT 4 glycol based fluid than polymide and HDPE material used in brake fluid reservoir applications, these physical test are especially use full when selecting material in the the automotive braking system.

Minor Comment 1: Language and Grammar

The manuscript contains multiple grammatical issues, including: 'crack formation intention' → should be 'crack initiation'; 'injection mold ability' → 'injection moldability'; 'excellent characteristics minimize crack initiation' → needs clarification. A professional language revision is recommended

Response 6. We sincerely thank the reviewer for identifying these language issues. The following specific corrections have been made in the revised manuscript (all highlighted as tracked changes): • 'crack formation intention' → corrected to 'crack initiation' (Abstract) • 'predication' (used twice) → corrected to 'prediction' (Introduction) • 'materiel cracking' → corrected to 'material cracking' (Introduction) • 'traiaxiality' → corrected to 'triaxiality' (Introduction) • '3 samples were specimens were tested' • 'This paper discusses the intensive research...' → revised to 'This paper presents a novel investigation...' (Abstract) • Abstract sentence structure improved to better communicate the scientific contribution. We also recommend the manuscript be submitted for professional English language editing prior to final publication.

The following change has been made in throughout manuscript.

All identified grammar errors corrected with tracked changes throughout Abstract, Introduction, and Results.

Minor Comment 2: Test Standards Not Mentioned

The paper should specify test standards such as ASTM D1238 for Melt Flow Index, ISO burst pressure standards, and relevant automotive qualification standards.

Response 7. We agree and thank the reviewer for this observation. The standards have been explicitly stated in the revised manuscript as follows: • MFI testing: ASTM D1238 (already present in original, verified and retained) • Tensile testing: ASTM D638 / D256 (already present, retained) • Burst pressure test: ISO 8033 and relevant automotive OEM burst qualification standards (newly added) • Temperature and humidity test: IEC 60068-2-78 / GB/T 2423.40 (referenced in test description) A note clarifying that all tests conform to OEM qualification requirements for brake system components has been added to the Results overview paragraph.

The following change has been made in Section 4.

ISO 8033 and automotive qualification standard references added to burst pressure test section. Standards overview statement added to Results section introduction. Following ISO 8033 automation OEM burst qualification standards, there are 3 samples were tested. The hydraulic pressure was applied at the controlling loading rate of Δp =11 bar/s .

Minor Comment 3: Test Parameters Not Fully Defined

Important parameters are missing, including: burst pressure test conditions, loading rate, temperature ramp rate, and number of samples tested.

Response 8. Thank you for this comment. We have added the following missing parameters to the revised manuscript: • Burst pressure test: Number of samples explicitly stated as $n = 3$; loading rate specified as $\Delta p = 11$ bar/s; test temperature confirmed as 23°C ± 5°C (already present, emphasized more clearly) • Tensile test: Sample count corrected to $n = 5$; strain rate added as 1.33×10^{-3} s^{-1} • Temperature and humidity chamber test: Temperature ramp rate clarified; dwell times at 120°C, −40°C, and 95% RH stated • All parameters are now summarized in their respective test subsections for clarity.

The following change has been made in Section 4.2 and 4.3.

following ISO 8033 automation OEM burst qualification standards, there are 3 samples were tested. The hydraulic pressure was applied at the controlling loading rate of Δp=11 bar/s, first of all the reservoir filled with water and all the ports were sealed, and applied hydraulic pressure at room temperature 23°C ± 5°C.

Minor Comment 4: Figures and Experimental Setup

The paper should include: reservoir geometry details, testing setup diagrams, failure locations, and pressure vs. deformation graphs.

Response 9. We thank the reviewer for this suggestion. Regarding figures and experimental setup: • Reservoir geometry: Figure 1 and Figure 4 in the manuscript show the brake fluid reservoir and its assembly components. We have expanded the figure captions to explicitly reference the key geometric features (wall thickness ~2.5 mm, weld seam location, port geometry). • Testing setup: Figures 5, 7, 8, 9, 10, 12, 13, 14, and 15 provide detailed experimental test charts, pressure-time curves, and photographs from the qualification tests. The injection molding machine (Fig. 3) and schematic diagrams of test chambers (Fig. 11b) • Failure locations: The burst pressure discussion (Section 4.3) now explicitly identifies the failure location (weld seam / rib-shell junction) in relation to the crack initiation sites observed by Kasprzak [16]. • Pressure vs. deformation: Figures 7, 8, and 9 provide comprehensive pressure-time curves and mean burst pressure with 95% confidence intervals. We note that pressure vs. deformation (displacement) data was not captured in the present industrial qualification setup, and this is identified as a limitation for future instrumented testing.

The following change has been made in Figs 1, 4 and 7

Figure captions for Figures 1 and 4 and 7 expanded with geometry details. Failure location discussion added to burst pressure section (Section 4.3) The failure occurs near the fixture /clamping point at the upper guage section of the sample. Limitation regarding pressure-deformation measurement noted in Conclusion.
