# Supplementary material for: Investigation of crack initiation, and mechanical behavior of polypropylene and polymer-based composites under plastic deformation in automotive brake fluid reservoirs
Source: PLoS One. 2026 Sep 18;21(9):e0352763. doi: 10.1371/journal.pone.0352763 (PMC13588379; doi:10.1371/journal.pone.0352763)
Supplement: Supplementary file 2 [file pone.0352763.s001.docx]

Review has been completed on your paper “Investigation of Crack Initiation, and Mechanical Behavior of Polypropylene and Polymer-Based Composites under Plastic Deformation in Automotive Brake Fluid Reservoirs”.

**General Assessment:** The work combines material characterization and component-level validation tests. Use of multiple industrial qualification tests provides practical insights. The application focus on automotive brake fluid reservoirs gives engineering relevance. However, several aspects reduce the claimed novelty: Polypropylene in brake fluid reservoirs is well established, Testing methods are standard industrial validation tests, Stress triaxiality analysis is not clearly developed,

Kindly add and update the paper with following points in the revised paper,

**Recommendation: Major Revision**

**Critical Reviewer Comments**

Major Comments

1. Weak Scientific Contribution

The manuscript mainly reports standard validation tests commonly used in automotive component qualification. The novelty and scientific contribution are limited.

The authors should clearly explain:

- What new scientific knowledge is generated?
- How does this work advance polymer fracture mechanics or material behavior understanding?

2. Triaxiality Analysis is Not Clearly Presented

The paper claims investigation of stress triaxiality, but:

- No triaxiality equation is presented
- No stress-state analysis is shown
- No fracture locus or ductile failure model is discussed

If triaxiality is a core part of the study, the paper should include:

- definition of stress triaxiality
- stress triaxiality calculation
- relation to crack initiation.

3. Lack of Mechanical Testing Data

The paper discusses mechanical behavior but does not report:

- tensile tests
- stress–strain curves
- yield strength
- fracture strain
- strain rate effects

These are essential for understanding polymer deformation behavior.

4. Crack Initiation Investigation is Insufficient

The paper claims to investigate crack initiation but provides:

- no microscopic fracture analysis
- no crack propagation images
- no fracture mechanics approach

Crack initiation studies typically include:

- SEM fracture surface analysis
- notch sensitivity testing
- fatigue crack growth.

5. Absence of Comparative Study

The research focuses only on SABIC 83MF10 polypropylene.

However, the study would be significantly stronger if it compared:

- PP vs reinforced PP
- PP vs polyamide
- PP vs HDPE.

3. Minor Comments

1. Language and Grammar

The manuscript contains multiple grammatical issues such as:

- “crack formation intention” → should be crack initiation
- “injection mold ability” → injection moldability
- “excellent characteristics minimize crack initiation” → needs clarification.

A professional language revision is recommended.

2. Test Standards Not Mentioned

The paper should specify standards such as:

- ASTM D1238 for Melt Flow Index
- ISO burst pressure standards
- automotive qualification standards

3. Test Parameters Not Fully Defined

Important parameters missing:

- burst pressure test conditions
- loading rate
- temperature ramp rate
- number of samples tested.

4. Figures and Experimental Setup

The paper should include:

- reservoir geometry
- testing setup diagrams
- failure locations
- pressure vs deformation graphs.
