# Supplementary material for: Investigation of crack initiation, and mechanical behavior of polypropylene and polymer-based composites under plastic deformation in automotive brake fluid reservoirs
Source: PLoS One. 2026 Sep 18;21(9):e0352763. doi: 10.1371/journal.pone.0352763 (PMC13588379; doi:10.1371/journal.pone.0352763)
Supplement: Peer review history

# Peer review: Decision Letter 0

**DOI:** 10.1371/journal.pone.0352763.r001

**Authors:** S. Shanmugan

25 Mar 2026

Dear Dr. Hussain,

Thank you for submitting your manuscript to PLOS ONE. After careful consideration, we feel that it has merit but does not fully meet PLOS ONE’s publication criteria as it currently stands. Therefore, we invite you to submit a revised version of the manuscript that addresses the points raised during the review process.

Please submit your revised manuscript by May 09 2026 11:59PM. If you will need more time than this to complete your revisions, please reply to this message or contact the journal office at plosone@plos.org. When you're ready to submit your revision, log on to https://www.editorialmanager.com/pone/ and select the 'Submissions Needing Revision' folder to locate your manuscript file.

- A letter that responds to each point raised by the academic editor and reviewer(s). You should upload this letter as a separate file labeled 'Response to Reviewers'.
- A marked-up copy of your manuscript that highlights changes made to the original version. You should upload this as a separate file labeled 'Revised Manuscript with Track Changes'.
- An unmarked version of your revised paper without tracked changes. You should upload this as a separate file labeled 'Manuscript'.

If you would like to make changes to your financial disclosure, please include your updated statement in your cover letter. Guidelines for resubmitting your figure files are available below the reviewer comments at the end of this letter.

If applicable, we recommend that you deposit your laboratory protocols in protocols.io to enhance the reproducibility of your results. Protocols.io assigns your protocol its own identifier (DOI) so that it can be cited independently in the future. For instructions see: https://journals.plos.org/plosone/s/submission-guidelines#loc-laboratory-protocols. Additionally, PLOS ONE offers an option for publishing peer-reviewed Lab Protocol articles, which describe protocols hosted on protocols.io. Read more information on sharing protocols at https://plos.org/protocols?utm_medium=editorial-email&utm_source=authorletters&utm_campaign=protocols.

As the corresponding author, your ORCID iD is verified in the submission system and will appear in the published article. PLOS supports the use of ORCID, and we encourage all coauthors to register for an ORCID iD and use it as well. Please encourage your coauthors to verify their ORCID iD within the submission system before final acceptance, as unverified ORCID iDs will not appear in the published article. *Only*  the individual author can complete the verification step; PLOS staff *cannot*  verify ORCID iDs on behalf of authors.

We look forward to receiving your revised manuscript.

Kind regards,

S. Shanmugan, PhD

Academic Editor

PLOS One

**Journal Requirements:**

When submitting your revision, we need you to address these additional requirements.

1. Please ensure that your manuscript meets PLOS ONE's style requirements, including those for file naming. The PLOS ONE style templates can be found at

https://journals.plos.org/plosone/s/file?id=wjVg/PLOSOne_formatting_sample_main_body.pdf and

https://journals.plos.org/plosone/s/file?id=ba62/PLOSOne_formatting_sample_title_authors_affiliations.pdf

2. Please update your submission to use the PLOS LaTeX template. The template and more information on our requirements for LaTeX submissions can be found at http://journals.plos.org/plosone/s/latex.

3. We note that the grant information you provided in the ‘Funding Information’ and ‘Financial Disclosure’ sections do not match.

When you resubmit, please ensure that you provide the correct grant numbers for the awards you received for your study in the ‘Funding Information’ section.

4. Thank you for stating the following in the Financial Disclosure section:

“Zhejiang Qiaoshi Industry Co., Ltd. pay funding”

We note that one or more of the authors are employed by a commercial company:

- Zhejiang Qiaoshi Industry Co., Ltd.,
- Ningbo Qiaoshi Rubber & Plastics Co., Ltd.,

1. Please provide an amended Funding Statement declaring this commercial affiliation, as well as a statement regarding the Role of Funders in your study. If the funding organization did not play a role in the study design, data collection and analysis, decision to publish, or preparation of the manuscript and only provided financial support in the form of authors' salaries and/or research materials, please review your statements relating to the author contributions, and ensure you have specifically and accurately indicated the role(s) that these authors had in your study. You can update author roles in the Author Contributions section of the online submission form.

Please also include the following statement within your amended Funding Statement.

“The funder provided support in the form of salaries for authors [insert relevant initials], but did not have any additional role in the study design, data collection and analysis, decision to publish, or preparation of the manuscript. The specific roles of these authors are articulated in the ‘author contributions’ section.”

If your commercial affiliation did play a role in your study, please state and explain this role within your updated Funding Statement.

2. Please also provide an updated Competing Interests Statement declaring this commercial affiliation along with any other relevant declarations relating to employment, consultancy, patents, products in development, or marketed products, etc.

Within your Competing Interests Statement, please confirm that this commercial affiliation does not alter your adherence to all PLOS ONE policies on sharing data and materials by including the following statement: "This does not alter our adherence to  PLOS ONE policies on sharing data and materials.” (as detailed online in our guide for authors http://journals.plos.org/plosone/s/competing-interests) . If this adherence statement is not accurate and  there are restrictions on sharing of data and/or materials, please state these. Please note that we cannot proceed with consideration of your article until this information has been declared.

Please include both an updated Funding Statement and Competing Interests Statement in your cover letter. We will change the online submission form on your behalf.

5. Please provide a complete Data Availability Statement in the submission form, ensuring you include all necessary access information or a reason for why you are unable to make your data freely accessible. If your research concerns only data provided within your submission, please write "All data are in the manuscript and/or supporting information files" as your Data Availability Statement.

6. Please amend the manuscript submission data (via Edit Submission) to include authors:

- Liang Jian Chun
- Ying Quanjia
- Yang Bin
- Zhang Debing
- Zhou Sen
- Asif Raza d
- Malik Haris d
- Kefu Wang

7. Please amend either the abstract on the online submission form (via Edit Submission) or the abstract in the manuscript so that they are identical.

8. Please ensure that you refer to Figure 12 in your text as, if accepted, production will need this reference to link the reader to the figure.

9. We note that Figures 1, 4, 10, 12, and 13 in your submission contain copyrighted images. All PLOS content is published under the Creative Commons Attribution License (CC BY 4.0), which means that the manuscript, images, and Supporting Information files will be freely available online, and any third party is permitted to access, download, copy, distribute, and use these materials in any way, even commercially, with proper attribution. For more information, see our copyright guidelines: http://journals.plos.org/plosone/s/licenses-and-copyright.

We require you to either (1) present written permission from the copyright holder to publish these figures specifically under the CC BY 4.0 license, or (2) remove the figures from your submission:

1. You may seek permission from the original copyright holder of Figure 1, 4, 10, 12, and 13 to publish the content specifically under the CC BY 4.0 license.

We recommend that you contact the original copyright holder with the Content Permission Form (http://journals.plos.org/plosone/s/file?id=7c09/content-permission-form.pdf) and the following text:

“I request permission for the open-access journal PLOS ONE to publish XXX under the Creative Commons Attribution License (CCAL) CC BY 4.0 (http://creativecommons.org/licenses/by/4.0/). Please be aware that this license allows unrestricted use and distribution, even commercially, by third parties. Please reply and provide explicit written permission to publish XXX under a CC BY license and complete the attached form.”

Please upload the completed Content Permission Form or other proof of granted permissions as an "Other" file with your submission.

In the figure caption of the copyrighted figure, please include the following text: “Reprinted from [ref] under a CC BY license, with permission from [name of publisher], original copyright [original copyright year].”

2. If you are unable to obtain permission from the original copyright holder to publish these figures under the CC BY 4.0 license or if the copyright holder’s requirements are incompatible with the CC BY 4.0 license, please either i) remove the figure or ii) supply a replacement figure that complies with the CC BY 4.0 license. Please check copyright information on all replacement figures and update the figure caption with source information. If applicable, please specify in the figure caption text when a figure is similar but not identical to the original image and is therefore for illustrative purposes only.

10. If the reviewer comments include a recommendation to cite specific previously published works, please review and evaluate these publications to determine whether they are relevant and should be cited. There is no requirement to cite these works unless the editor has indicated otherwise.

**Additional Editor Comments:**

Dear Editor in chief Journal of PLOS One

I have read whole the manuscript and the below sentences are my idea and comments regarding the authors’ researcher. In my point of view some points were missed:

1. The written abstract must be rewritten and more obtained quantitative results and produced samples should be added.

2. What is the novelty of this research? There are many research similarities to this project? Does the introduction adequately highlight the novelty topic?

3. The state of art manuscript regarding to the subject must be added as literature. Are the cited references sufficiently recent and comprehensive to cover the topic?

4. Its recommended that the literature be similar to the manuscript subject, for examples using manuscript with the same base material. How does the study differentiate its approach from previous work ?

5. Please add more explain about the obtained results.

6. Please change the figures by better and more quality images.

7. Title/abstract claim "crack initiation" analysis, but no fracture mechanics, notched tests, or triaxiality data. Revise claims or add material-level crack tests (SEM, DIC).

8. Burst tests show no pre-failure cracks up to ~20–22 bar. Provide high-speed images/SEM fractographs showing crack path to support "no premature cracks".

9. Triaxiality repeatedly mentioned (citing Kasprzak 2024) but no values, stress-state calc, or PEEK/TRIAX correlation. Add FEA triaxiality maps or remove claims.

10. Only SABIC 83MF10 tested—no comparison with other PP grades or glass-filled variants. Justify choice and provide competitor baselines.

11. Burst pressures 20–22 bar reported—no OEM spec or safety margin stated. Cite target (e.g., SAE J2053) and margin.

12. Air leakage holds 500–700 kPa—no leak rate or pass/fail criterion. Specify standard and quantitative limit (e.g., <1 kPa/min).

13. Humidity test (120°C/-40°C, 95% RH, 24 h) claims "no deviation". Provide pre/post dimensions and post-exposure burst data.

14. Tilt/alarm activates at 0.53° with 0.004 V. Clarify sensor type, fluid level, and provide tilt vs. voltage curve with threshold.

15. Volume test checks "deviation"—no quantitative results. Provide measured vs. nominal volumes and tolerances.

16. No material-level tests (tensile, flexural, impact) on PP samples from reservoir. Add ASTM D638/D256 data to support "mechanical behavior".

17. No weld seam characterization (hot-plate strength, microstructure). Provide weld interface SEM or lap-shear data.

18. No long-term aging (brake fluid immersion, vibration, extended thermal cycling). Justify why short tests suffice for "long-term reliability".

19. Figures lack clear labels/scales/legends (e.g., Fig. 7 pressure-time unclear). Improve captions and formatting.

20. Most tests lack statistics (mean ± SD, n, CI). Provide replicates and p-values where relevant.

21. Will you supply supplementary: (a) raw burst/air data, (b) dimensional pre/post-humidity, (c) tilt-voltage curves, (d) weld/fracture SEMs, (e) SABIC datasheet, (f) spec compliance summary?

[Note: HTML markup is below. Please do not edit.]

Reviewers' comments:

Reviewer's Responses to Questions

**Comments to the Author**

1. Is the manuscript technically sound, and do the data support the conclusions?

Reviewer #1: Partly

Reviewer #2: Partly

**********

2. Has the statistical analysis been performed appropriately and rigorously?

Reviewer #1: Yes

Reviewer #2: No

**********

3. Have the authors made all data underlying the findings in their manuscript fully available?

The PLOS Data policy

Reviewer #1: Yes

Reviewer #2: No

**********

4. Is the manuscript presented in an intelligible fashion and written in standard English?

Reviewer #1: Yes

Reviewer #2: No

**********

Reviewer #1: Dear Editor in chief Journal of PLOS One

I have read whole the manuscript and the below sentences are my idea and comments regarding the authors’ researcher. In my point of view some points were missed:

1. The written abstract must be rewritten and more obtained quantitative results and produced samples should be added.

2. What is the novelty of this research? There are many research similarities to this project? Does the introduction adequately highlight the novelty topic?

3. The state of art manuscript regarding to the subject must be added as literature. Are the cited references sufficiently recent and comprehensive to cover the topic?

4. Its recommended that the literature be similar to the manuscript subject, for examples using manuscript with the same base material. How does the study differentiate its approach from previous work ?

5. Please add more explain about the obtained results.

6. Please change the figures by better and more quality images.

7. Title/abstract claim "crack initiation" analysis, but no fracture mechanics, notched tests, or triaxiality data. Revise claims or add material-level crack tests (SEM, DIC).

8. Burst tests show no pre-failure cracks up to ~20–22 bar. Provide high-speed images/SEM fractographs showing crack path to support "no premature cracks".

9. Triaxiality repeatedly mentioned (citing Kasprzak 2024) but no values, stress-state calc, or PEEK/TRIAX correlation. Add FEA triaxiality maps or remove claims.

10. Only SABIC 83MF10 tested—no comparison with other PP grades or glass-filled variants. Justify choice and provide competitor baselines.

11. Burst pressures 20–22 bar reported—no OEM spec or safety margin stated. Cite target (e.g., SAE J2053) and margin.

12. Air leakage holds 500–700 kPa—no leak rate or pass/fail criterion. Specify standard and quantitative limit (e.g., <1 kPa/min).

13. Humidity test (120°C/-40°C, 95% RH, 24 h) claims "no deviation". Provide pre/post dimensions and post-exposure burst data.

14. Tilt/alarm activates at 0.53° with 0.004 V. Clarify sensor type, fluid level, and provide tilt vs. voltage curve with threshold.

15. Volume test checks "deviation"—no quantitative results. Provide measured vs. nominal volumes and tolerances.

16. No material-level tests (tensile, flexural, impact) on PP samples from reservoir. Add ASTM D638/D256 data to support "mechanical behavior".

17. No weld seam characterization (hot-plate strength, microstructure). Provide weld interface SEM or lap-shear data.

18. No long-term aging (brake fluid immersion, vibration, extended thermal cycling). Justify why short tests suffice for "long-term reliability".

19. Figures lack clear labels/scales/legends (e.g., Fig. 7 pressure-time unclear). Improve captions and formatting.

20. Most tests lack statistics (mean ± SD, n, CI). Provide replicates and p-values where relevant.

21. Will you supply supplementary: (a) raw burst/air data, (b) dimensional pre/post-humidity, (c) tilt-voltage curves, (d) weld/fracture SEMs, (e) SABIC datasheet, (f) spec compliance summary?

Reviewer #2: Review has been completed on your paper “Investigation of Crack Initiation, and Mechanical Behavior of Polypropylene and Polymer-Based Composites under Plastic Deformation in Automotive Brake Fluid Reservoirs”.

General Assessment: The work combines material characterization and component-level validation tests. Use of multiple industrial qualification tests provides practical insights. The application focus on automotive brake fluid reservoirs gives engineering relevance. However, several aspects reduce the claimed novelty: Polypropylene in brake fluid reservoirs is well established, Testing methods are standard industrial validation tests, Stress triaxiality analysis is not clearly developed,

Kindly add and update the paper with following points in the revised paper,

Recommendation: Major Revision

Critical Reviewer Comments

Major Comments

1. Weak Scientific Contribution

The manuscript mainly reports standard validation tests commonly used in automotive component qualification. The novelty and scientific contribution are limited.

The authors should clearly explain:

• What new scientific knowledge is generated?

• How does this work advance polymer fracture mechanics or material behavior understanding?

2. Triaxiality Analysis is Not Clearly Presented

The paper claims investigation of stress triaxiality, but:

• No triaxiality equation is presented

• No stress-state analysis is shown

• No fracture locus or ductile failure model is discussed

If triaxiality is a core part of the study, the paper should include:

• definition of stress triaxiality

• stress triaxiality calculation

• relation to crack initiation.

3. Lack of Mechanical Testing Data

The paper discusses mechanical behavior but does not report:

• tensile tests

• stress–strain curves

• yield strength

• fracture strain

• strain rate effects

These are essential for understanding polymer deformation behavior.

4. Crack Initiation Investigation is Insufficient

The paper claims to investigate crack initiation but provides:

• no microscopic fracture analysis

• no crack propagation images

• no fracture mechanics approach

Crack initiation studies typically include:

• SEM fracture surface analysis

• notch sensitivity testing

• fatigue crack growth.

5. Absence of Comparative Study

The research focuses only on SABIC 83MF10 polypropylene.

However, the study would be significantly stronger if it compared:

• PP vs reinforced PP

• PP vs polyamide

• PP vs HDPE.

3. Minor Comments

1. Language and Grammar

The manuscript contains multiple grammatical issues such as:

• “crack formation intention” → should be crack initiation

• “injection mold ability” → injection moldability

• “excellent characteristics minimize crack initiation” → needs clarification.

A professional language revision is recommended.

2. Test Standards Not Mentioned

The paper should specify standards such as:

• ASTM D1238 for Melt Flow Index

• ISO burst pressure standards

• automotive qualification standards

3. Test Parameters Not Fully Defined

Important parameters missing:

• burst pressure test conditions

• loading rate

• temperature ramp rate

• number of samples tested.

4. Figures and Experimental Setup

The paper should include:

• reservoir geometry

• testing setup diagrams

• failure locations

• pressure vs deformation graphs.

**********

what does this mean?). If published, this will include your full peer review and any attached files.

If you choose “no”, your identity will remain anonymous but your review may still be made public.

**Do you want your identity to be public for this peer review?**  For information about this choice, including consent withdrawal, please see our Privacy Policy

Reviewer #1: No

Reviewer #2: No

**********

[NOTE: If reviewer comments were submitted as an attachment file, they will be attached to this email and accessible via the submission site. Please log into your account, locate the manuscript record, and check for the action link "View Attachments". If this link does not appear, there are no attachment files.]

To ensure your figures meet our technical requirements, please review our figure guidelines: https://journals.plos.org/plosone/s/figures

You may also use PLOS’s free figure tool, NAAS, to help you prepare publication quality figures: https://journals.plos.org/plosone/s/figures#loc-tools-for-figure-preparation.

NAAS will assess whether your figures meet our technical requirements by comparing each figure against our figure specifications.

Attachment Submitted filename: Review Report.docx

# Author comment: Author response to Decision Letter 1

**DOI:** 10.1371/journal.pone.0352763.r002

19 May 2026

Thank you for your valuable comments and suggestions. We have revised the manuscript carefully and addressed all comments point by point in the attached response letter. The changes have been incorporated into the revised manuscript, and a track-changes version has also been provided.

Attachment Submitted filename: Responce to Reviewers.pdf

# Peer review: Decision Letter 1

**DOI:** 10.1371/journal.pone.0352763.r003

**Authors:** Yasir Nawab

16 Jun 2026

Investigation of Crack Initiation, and Mechanical Behavior of Polypropylene and Polymer-Based Composites under Plastic Deformation in Automotive Brake Fluid Reservoirs

PONE-D-26-03733R1

Dear Dr. Hussain,

We’re pleased to inform you that your manuscript has been judged scientifically suitable for publication and will be formally accepted for publication once it meets all outstanding technical requirements.

Within one week, you’ll receive an e-mail detailing the required amendments. When these have been addressed, you’ll receive a formal acceptance letter and your manuscript will be scheduled for publication.

An invoice will be generated when your article is formally accepted. Please note, if your institution has a publishing partnership with PLOS and your article meets the relevant criteria, all or part of your publication costs will be covered. Please make sure your user information is up-to-date by logging into Editorial Manager at Editorial Manager® and clicking the ‘Update My Information' link at the top of the page. For questions related to billing, please contact billing support.

If your institution or institutions have a press office, please notify them about your upcoming paper to help maximize its impact. If they’ll be preparing press materials, please inform our press team as soon as possible -- no later than 48 hours after receiving the formal acceptance. Your manuscript will remain under strict press embargo until 2 pm Eastern Time on the date of publication. For more information, please contact onepress@plos.org.

Kind regards,

Yasir Nawab, PhD

Academic Editor

PLOS One

Additional Editor Comments (optional):

Reviewers' comments:

Reviewer's Responses to Questions

**Comments to the Author**

Reviewer #1: (No Response)

Reviewer #2: All comments have been addressed

**********

2. Is the manuscript technically sound, and do the data support the conclusions?

Reviewer #1: (No Response)

Reviewer #2: Yes

**********

3. Has the statistical analysis been performed appropriately and rigorously?

Reviewer #1: (No Response)

Reviewer #2: Yes

**********

4. Have the authors made all data underlying the findings in their manuscript fully available?

The PLOS Data policy

Reviewer #1: (No Response)

Reviewer #2: No

**********

5. Is the manuscript presented in an intelligible fashion and written in standard English?

Reviewer #1: (No Response)

Reviewer #2: Yes

**********

Reviewer #1: (No Response)

Reviewer #2: Review has been completed on your paper “Investigation of Crack Initiation, and Mechanical Behavior of Polypropylene and Polymer-Based Composites under Plastic Deformation in Automotive Brake Fluid Reservoirs”. Based on the response to queries in the review, I am pleased to accept the manuscript.

**********

what does this mean?). If published, this will include your full peer review and any attached files.

If you choose “no”, your identity will remain anonymous but your review may still be made public.

**Do you want your identity to be public for this peer review?**  For information about this choice, including consent withdrawal, please see our Privacy Policy

Reviewer #1: No

Reviewer #2: **Yes:**  Dr Imran Moulaalli Jamadar

**********

# Editor report: Acceptance letter

**DOI:** 10.1371/journal.pone.0352763.r004

**Authors:** Yasir Nawab

PONE-D-26-03733R1

PLOS One

Dear Dr. Hussain,

I'm pleased to inform you that your manuscript has been deemed suitable for publication in PLOS One. Congratulations! Your manuscript is now being handed over to our production team.

At this stage, our production department will prepare your paper for publication. This includes ensuring the following:

* All references, tables, and figures are properly cited

* All relevant supporting information is included in the manuscript submission,

* There are no issues that prevent the paper from being properly typeset

You will receive further instructions from the production team, including instructions on how to review your proof when it is ready. Please keep in mind that we are working through a large volume of accepted articles, so please give us a few days to review your paper and let you know the next and final steps.

Lastly, if your institution or institutions have a press office, please let them know about your upcoming paper now to help maximize its impact. If they'll be preparing press materials, please inform our press team within the next 48 hours. Your manuscript will remain under strict press embargo until 2 pm Eastern Time on the date of publication. For more information, please contact onepress@plos.org.

You will receive an invoice from PLOS for your publication fee after your manuscript has reached the completed accept phase. If you receive an email requesting payment before acceptance or for any other service, this may be a phishing scheme. Learn how to identify phishing emails and protect your accounts at https://explore.plos.org/phishing.

If we can help with anything else, please email us at customercare@plos.org.

Thank you for submitting your work to PLOS One and supporting open access.

Kind regards,

PLOS One Editorial Office Staff

on behalf of

Dr. Yasir Nawab

%CORR_ED_EDITOR_ROLE%

PLOS One
